# Green multicomponent synthesis of pyrano[2,3-*c*]pyrazole derivatives: current insights and future directions

**DOI:** 10.1039/d3ra05570a

**Published:** 2023-10-02

**Authors:** Afrisham Ahmad, Sithara Rao, Nitinkumar S. Shetty

**Affiliations:** a Department of Chemistry, Manipal Institute of Technology, Manipal Academy of Higher Education Manipal Karnataka 576104 India nitin.shetty@manipal.edu

## Abstract

The past decade has witnessed significant progress in synthesizing structurally diverse and biologically relevant pyrano[2,3-*c*]pyrazole derivatives through the integration of green methodologies. This review summarizes the recent advances in the green multicomponent synthesis of pyrano[2,3-*c*]pyrazole and spiro-pyrano[2,3-*c*]pyrazole derivatives. These include the application of energy-efficient techniques such as microwave and ultrasound-assisted synthesis, benign catalysts and biodegradable composites, solvent selection with a focus on water as a renewable and non-toxic medium, and solvent-free conditions. The review consolidates the current knowledge and future research directions, providing a valuable resource for researchers dedicated to advancing green chemistry practices.

## Abstract 2

The past decade has witnessed significant progress in synthesizing structurally diverse and biologically relevant pyrano[2,3-*c*]pyrazole derivatives through the integration of green methodologies.

## Graphical abstract

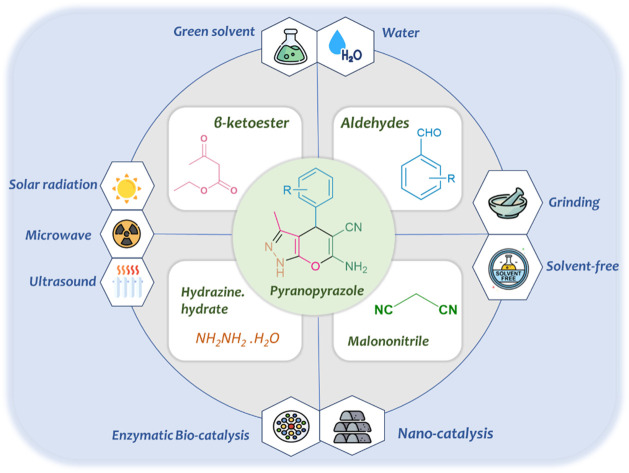

## 1 Introduction

In recent years, the field of organic synthesis has witnessed a remarkable paradigm shift towards sustainability and environmental consciousness. This transformation is illustrated by the emergence of green chemistry, which advocates for the development of eco-friendly and resource-efficient synthetic methodologies.^1,2^ Among the myriad reactions and strategies in organic synthesis, the multicomponent synthesis of heterocyclic compounds holds a prominent position due to its efficiency and versatility.^3^

**Fig. 1:**
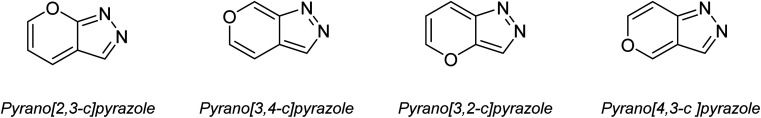
Isomers of pyranopyrazoles.

**Fig. 2:**
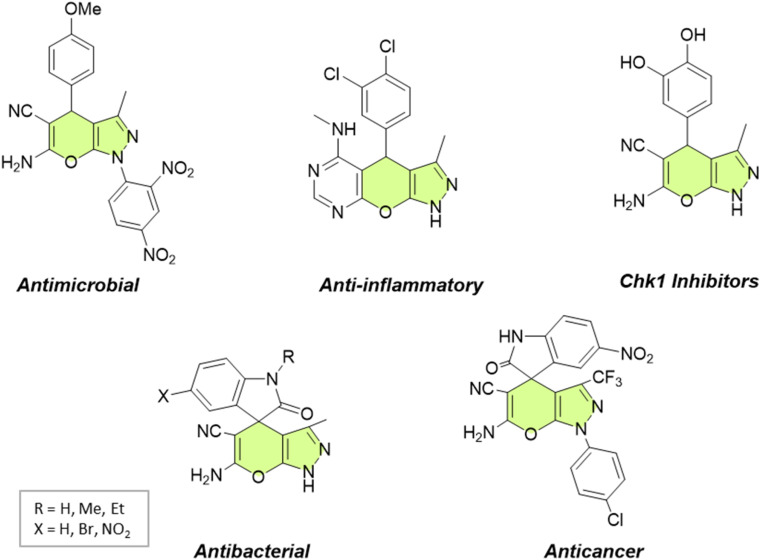
Some biologically active pyrano[2,3-*c*]pyrazoles.

Pyranopyrazoles, as a subclass of heterocycles, have garnered significant attention owing to their diverse structural significance and biological activities.^4–6^ These compounds are composed of fused pyran and pyrazole rings, existing in four distinct isomer arrangements: pyrano[2,3-*c*]pyrazole, pyrano[3,2-*c*]pyrazole, pyrano[3,4-*c*]pyrazole, and pyrano[4,3-*c*]pyrazole (Fig. 1). However, pyrano[2,3-*c*]pyrazoles are the most extensively investigated due to the biological significance of this isomer. These compounds have shown promising antimicrobial,^7,8^ anticancer,^9^ anti-inflammatory,^10^ and antiviral properties.^11^ Additionally, they exhibit the capability to potentially inhibit the activity of the human Chk1 kinase enzyme^12^ (Fig. 2). Their structural diversity allows for the modulation of activity by modifying different regions of the molecule, opening up possibilities for structure–activity relationship studies.^13^

The synthesis of pyrano[2,3-*c*]pyrazole has, undeniably, been a subject of considerable research efforts, yielding numerous methods and synthetic routes. Yet, the existing body of literature predominantly focuses on achieving high yields and product diversity, often overshadowing the critical importance of sustainability. Traditional methods for their synthesis often require multiple reactions and purification steps in harsh reaction conditions, such as high temperatures or strong acids, leading to low yields and potential side reactions. Furthermore, the use of toxic solvents, hazardous reagents, and high energy consumption contribute to environmental pollution, waste generation, and carbon emissions.^2^ While the exploration of various reaction pathways and synthetic strategies is undoubtedly essential, it is equally imperative to acknowledge the environmental impact of these processes. The past decade has witnessed significant progress in this area, with researchers developing innovative strategies and employing green principles to access pyranopyrazoles efficiently.^14,15^ Among the green techniques in organic chemistry are reactions involving solid-supported, bio- and asymmetric catalysis and synthesis,^16,17^ water and other green solvents,^2^ ionic liquids (ILs) or without solvents, microwave, ultrasound, ultraviolet (UV), and flow reactors.^18,19^

**Fig. 3:**
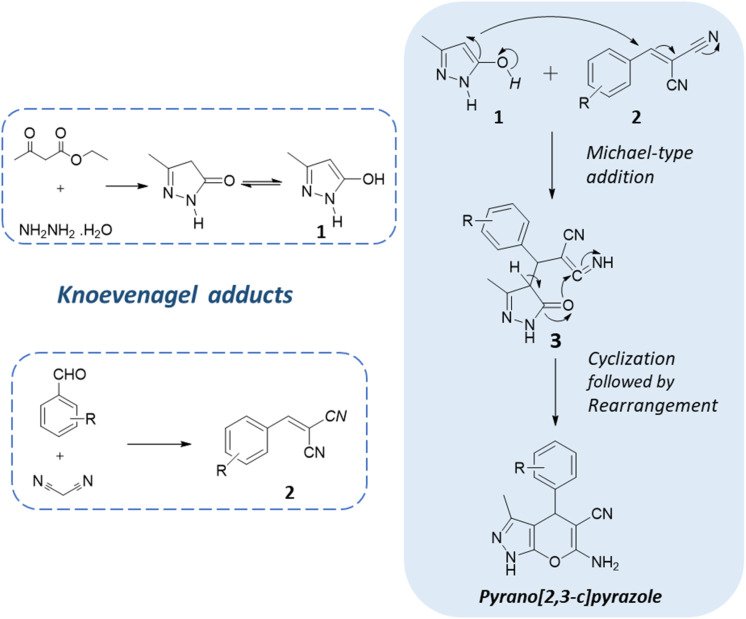
General reaction scheme for the one-pot multicomponent synthesis of pyrano[2,3-*c*]pyrazole.

One of the key strategies employed in the synthesis of pyranopyrazoles is multicomponent reactions. These are one-pot reactions that involve the sequential addition of multiple reagents and catalysts, enabling the rapid assembly of the target molecules in a single reaction vessel.^20,21^ The general reaction scheme of the one-pot multicomponent reaction of pyranopyrazoles typically involves an aldehyde, malononitrile, a β-ketoester/ethyl acetoacetate, hydrazine hydrate, and an appropriate catalyst or promoter.^22^ The reaction proceeds through a series of sequential transformations, including condensation, cyclization, and subsequent rearrangement, yielding the pyranopyrazole product (Fig. 3). MCRs often proceed under mild reaction conditions, minimizing the need for harsh reagents.^23,24^ They offer several advantages, including atom economy, step economy, and the simultaneous assembly of multiple building blocks and thus comply with the principles of green chemistry. Furthermore, the one-pot nature of this reaction reduces the number of purification steps required, minimizing potential side reactions and simplifying the overall synthetic process.^25,26^

Numerous preceding review articles have appropriately lauded the progress in pyranopyrazole synthesis through MCRs,^27,28^ showcasing ingenious strategies, high yields, and novel applications. In this landscape, our review article aims to stand apart by offering a fresh perspective on the synthesis of pyranopyrazoles through MCRs, one that prioritizes the principles of green chemistry.

In this comprehensive review article, we have meticulously examined a decade's worth of research papers, spanning from 2012 to 2023, in order to provide a holistic overview of the advancements made in the green multicomponent synthesis of pyrano[2,3-*c*]pyrazoles and spiro-pyrano[2,3-*c*]pyrazole derivatives. Our primary objective was to focus on research that not only explored various synthetic routes but also adhered to the fundamental principles of green chemistry. Instead of limiting our scope to a single green chemistry principle, we sought out studies that harmoniously integrated multiple eco-friendly strategies, for instance, the application of energy-efficient techniques such as microwave and ultrasound-assisted synthesis, catalyst design using environmentally friendly metals and biodegradable composites, solvent selection with a focus on water as a renewable and non-toxic medium, and solvent-free conditions. This discerning approach allowed us to select and showcase papers that exemplified the multifaceted nature of sustainable synthesis. In our review, we have placed particular emphasis on elucidating the key findings and novel methodologies outlined in these selected papers. Through this extensive exploration, we aim to offer readers a comprehensive and insightful understanding of the green multicomponent synthesis of pyranopyrazoles, while highlighting the pivotal role of sustainable chemistry in shaping the future of organic synthesis.

## 2 Physical methods

Energy inputs play a crucial role in organic synthesis, influencing reaction rates, yields, selectivity, and overall process efficiency. Conventional heating supplies the necessary energy to surmount activation barriers. However, to maintain a balanced energy system during prolonged reaction times, a cooling medium such as a water reflux condenser is essential for the efficient transfer of thermal energy.^29^ Reaction temperatures can be high, which may cause undesired side reactions that can be less sustainable compared to green approaches like microwave heating, ultrasound irradiation, concentrated solar radiation, *etc.* These alternative energy inputs are characterized by their potential to reduce energy consumption, minimize waste, and promote sustainable practices. They can also lead to shorter reaction times, higher yields, and improved product selectivity.^30^

### 2.1 Microwave-assisted technique

Microwave irradiation provides rapid and selective heating of reaction mixtures using electromagnetic waves. They accelerate reactions due to direct interaction with polar molecules and lead to shorter reaction times as well as increased yields making the process energy-efficient.^31,32^

**Scheme 1:**
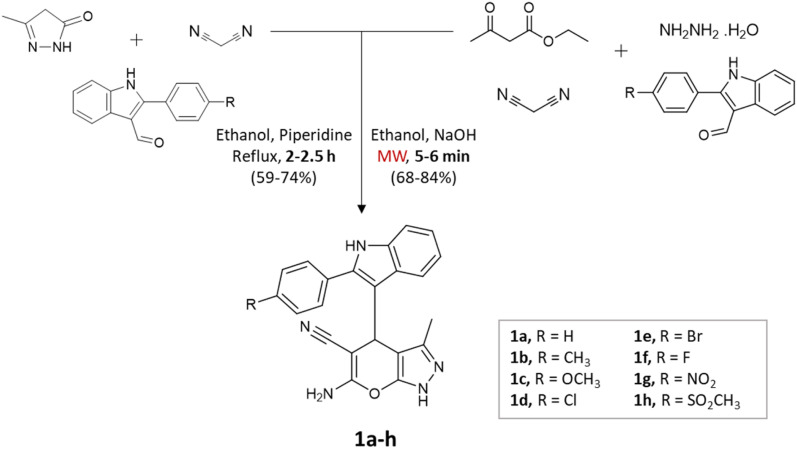
Synthesis of indol-3-yl substituted pyrano[2,3-*c*]pyrazoles using conventional and microwave-assisted methods.

Kathrotiya *et al.*^33^ synthesized a series of indol-3-yl substituted pyrano[2,3-*c*]pyrazoles using two different methods: a conventional three-component reaction under reflux conditions and a four-component reaction, with the assistance of microwave irradiation (Scheme 1). In the three-component reaction, 2-phenyl-1*H*-indole-3-carbaldehydes, malononitrile, and 3-methyl-1*H*-pyrazol-5(4*H*)-one were condensed in ethanol with piperidine. The reaction mixture was gradually heated and refluxed for 2–2.5 h. On the other hand, the four-component reaction involved the condensation of 2-phenyl-1*H*-indole-3-carbaldehydes, ethyl acetoacetate, malononitrile, and hydrazine hydrate in ethanol with NaOH as the catalyst. Microwave irradiation at an output power of 280 W was applied to the mixture for a period of 5–6 min. A comparative analysis of the two methods revealed that microwave irradiation proved to be more effective in accelerating the reactions.

**Scheme 2:**
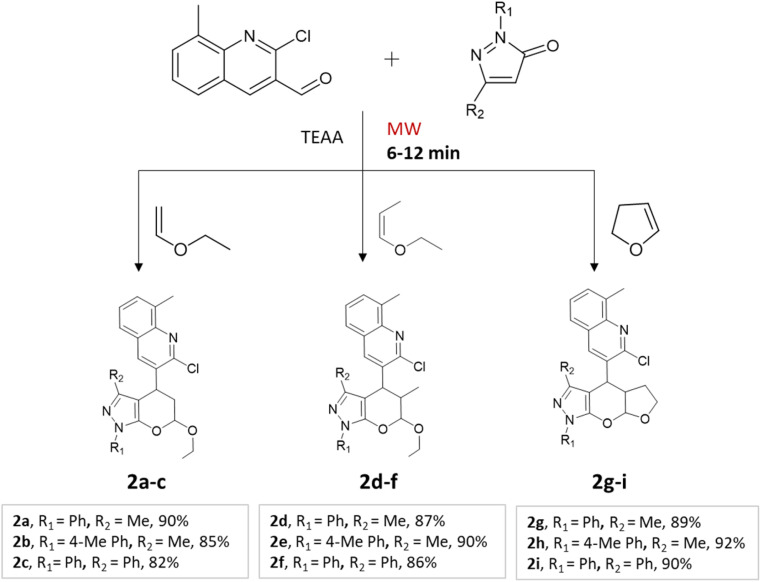
Synthesis of quinolylpyrano[2,3-*c*]pyrazoles using TEAA under microwave irradiation.

**Scheme 3:**
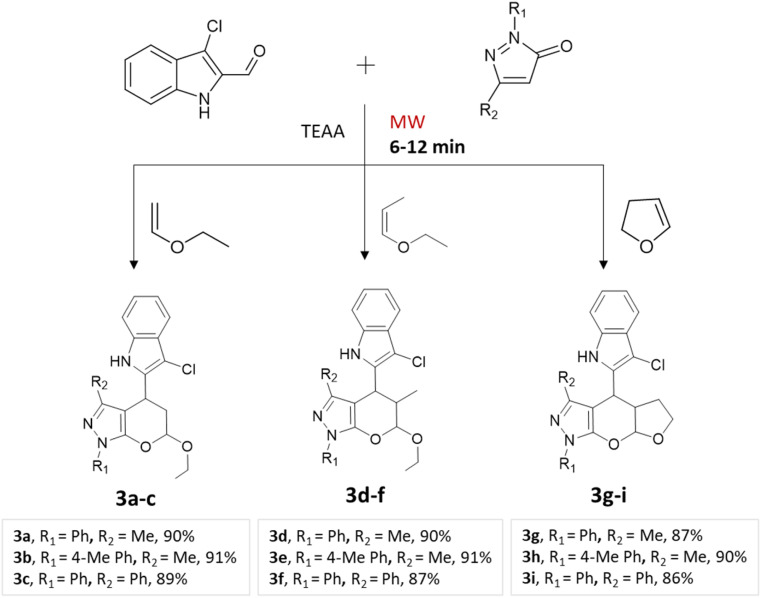
Synthesis of indolylpyrano[2,3-*c*]pyrazoles using TEAA under microwave irradiation.

A regio- and stereoselective method for synthesizing heteroaryl pyranopyrazoles was developed by J. Parmar and colleagues.^34^ The procedure involved a multi-component domino reaction using indole- or quinolcarbaldehyde, pyrazolone, and enol ethers in triethylammonium acetate (TEAA) under the influence of microwave irradiation (Schemes 2 and 3). To optimize the reaction conditions, different refluxing solvents (acetylene, xylene, toluene, TEAA) were used. It was found that ionic liquid TEAA as a reaction medium required no catalyst and yielded 88% of the desired products in 5 h. By employing microwave irradiation, the reaction time was further reduced to just 10 min.

**Scheme 4:**
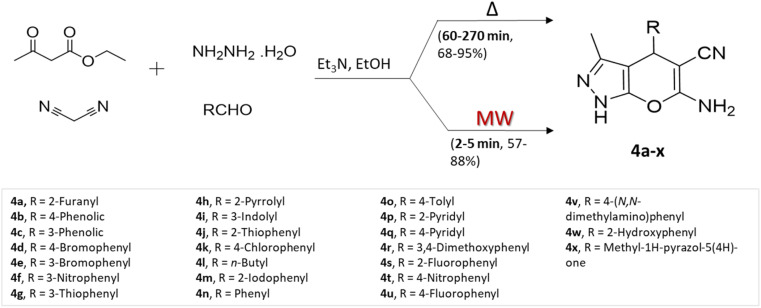
Synthesis of pyrano[2,3-*c*]pyrazoles using traditional heating and microwave-assisted techniques.

P. Shukla and colleagues^35^ prepared a range of pyrano[2,3-*c*]pyrazole using two different methods: conventional heating and microwave-assisted multicomponent approach, involving ethyl acetoacetate, hydrazine, malononitrile, and aldehydes using triethylamine base (Scheme 4). Assessing the two approaches according to yields obtained and reaction completion times, the researchers noted that although the conventional heating method yielded slightly superior results in terms of product yields, the microwave-assisted synthesis notably and substantially reduced reaction durations.

**Scheme 5:**
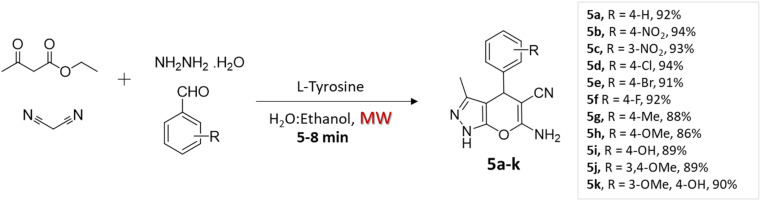
Synthesis of pyrano[2,3-*c*]pyrazoles using l-tyrosine under microwave irradiation.

Rupnar *et al.*^36^ employed the combination of microwave irradiation and an eco-friendly solvent (H_2_O–ethanol) to facilitate the synthesis of pyrano[2,3-*c*]pyrazole derivatives. This innovative approach involved the four-component condensation of acetoacetic ester, hydrazine hydride, aldehydes, and malononitrile, in the presence of l-tyrosine (Scheme 5).

**Scheme 6:**
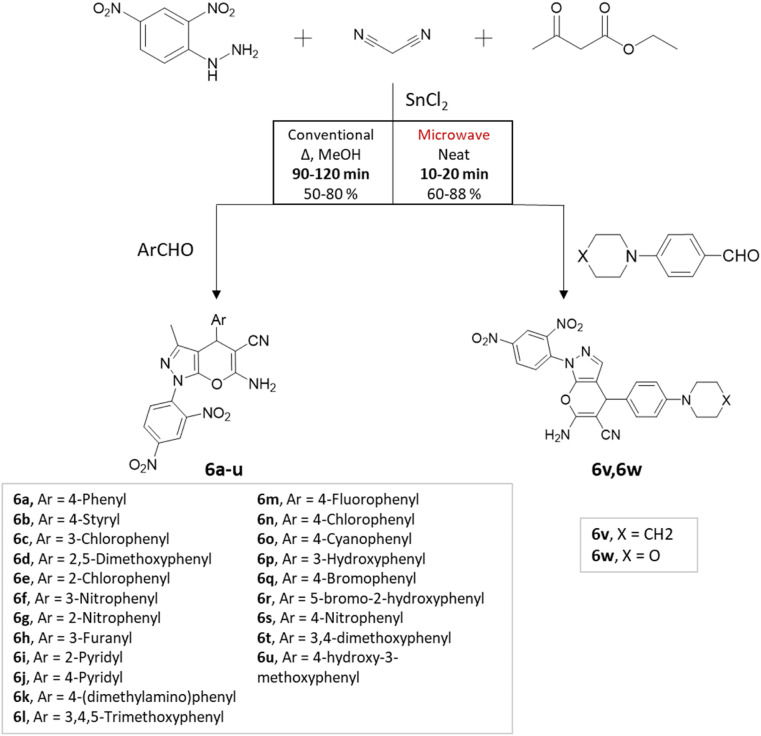
Pyrano[2,3-*c*] pyrazole synthesis using traditional heating and microwave-assisted techniques.

M. S. Vasava *et al.*^37^ successfully synthesized biologically active heterocyclic scaffolds based on pyrano[2,3-*c*]pyrazole using an MCR approach. Various substituted aldehyde derivatives were combined with 2,4-dinitrophenyl hydrazine, ethyl acetoacetate, and malononitrile using SnCl_2_ as the catalyst (Scheme 6). Two methods were compared: conventional heating and microwave irradiation. In the conventional method at 80 °C, the reaction took 1.4 h and resulted in an 80% yield. However, the microwave irradiation method produced the desired product in just 25 min, with an 88% yield.

**Scheme 7:**
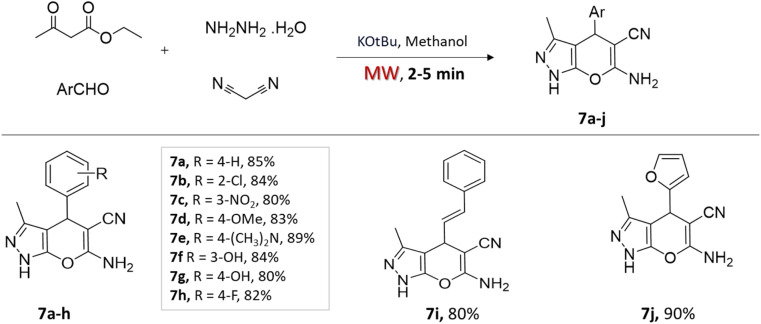
Synthesis of pyrano[2,3-*c*]pyrazoles using K-^*t*^BuO under microwave-assisted technique.

In a very recent study by Yallappa *et al.*,^38^ potassium *t*-butoxide, a base catalyst, was employed in a one-pot four-component approach to synthesize various 4*H*-pyrano[2,3-*c*]pyrazoles (Scheme 7). The condensation reaction involves a mixture of ethyl acetoacetate, hydrazine hydrate, malononitrile, and aromatic aldehydes in the methanol solvent with a catalytic amount of KO*t*Bu. Microwave irradiation led to faster reaction completion (less than 5 min) and excellent yields for the synthesized compounds compared to conventional stirring at room temperature (Table 1).

**Table 1:** A summary of microwave-assisted pyrano[2,3-*c*]pyrazole and spiropyrano[2,3-*c*] pyrazole synthesis

Product	Reactants	Catalyst	Solvent	Method employed	Reaction time	Yield	Ref.
Indol-3-yl substituted pyrano[2,3-*c*]pyrazoles	(a) Three-component system: 2-phenyl-1*H*-indole-3-carbaldehydes, malononitrile, and 3-methyl-1*H*-pyrazole-5(4*H*)-one	Piperidine	Ethanol	Conventional reflux	2–2.5 h	59–74%	Kathrotiya *et al.*^33^
	(b) Four-component system: 2-phenyl-1*H*-indole-3-carbaldehydes, ethyl acetoacetate, malononitrile, and hydrazine hydrate	NaOH		Microwave	5–6 min	68–84%	
Quinolylpyrano[2,3-*c*]pyrazoles and indolylpyrano[2,3-*c*]pyrazoles	Three-component system: indole- or quinol carbaldehyde, pyrazolone, and enol ethers	—	Triethyl-ammonium acetate (TEAA)	Microwave	6–12 min	82–92%	J. Parmar *et al.*^34^
Substituted pyrano[2,3-*c*]pyrazoles	Four-component system: acetoacetic ester, hydrazine hydride, aldehydes, and malononitrile	l-Tyrosine	H_2_O–ethanol	Microwave	5–8 min	86–94%	D. Rupnar *et al.*^36^
Pyrano[2,3-*c*]pyrazoles	Four-component system: ethyl acetoacetate, hydrazine, malononitrile, and aldehydes	Triethylamine	Ethanol	Conventional reflux	1–4.5 h	68–95%	P. Shukla *et al.*^35^
				Microwave	2–5 min	57–88%	
Pyrano[2,3-*c*]pyrazoles	Four-component system: substituted aldehyde, 2,4-dinitrophenyl hydrazine, ethyl acetoacetate, and malononitrile	SnCl_2_	Methanol	Conventional reflux	1.5–2 h	50–80%	S. Vasava *et al.*^37^
			Solvent-free	Microwave	10–20 min	60–88%	
4*H*-Pyrano[2,3-*c*] pyrazoles	Four-component system: ethyl acetoacetate, hydrazine hydrate, malononitrile, and aromatic aldehydes	Potassium *t*-butoxide (base)	Methanol	Microwave	2–5 min	80–90%	Yallappa *et al.*^38^

While microwave-assisted heating significantly reduces reaction times, most reactions still yielded comparable results to those achieved with conventional reflux heating. It is worth noting that there were instances where the yield was not as substantial. Hence, as a note to future research, it is essential to consider all factors, as they may not depend solely on the heating technique, but also on variables such as substituents, solvents, and catalysts.

### 2.2 Concentrated solar radiation technique

The concentrated solar radiation method involves focusing sunlight using a solar collector concentrated onto a reaction vessel with optical instruments and a temperature sensor to achieve the desired temperature for a chemical reaction. However, there is limited control over reaction temperature owing to the dependence on sunlight availability. These are also specific to certain reactions and geographical locations.^39,40^

**Scheme 8:**
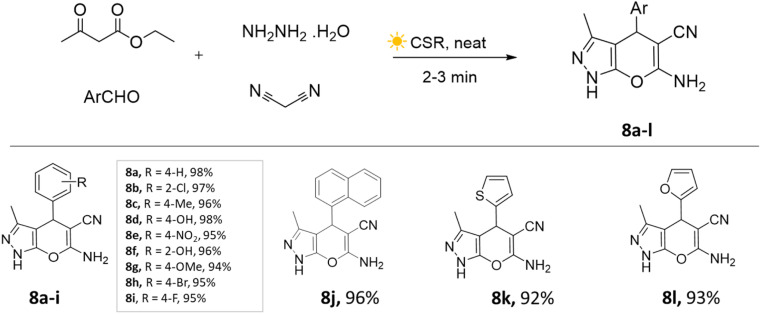
Synthesis of pyrano[2,3-*c*]pyrazoles using CSR technique.

Yatin U. Gadkari *et al.*,^41^ showcased the utilization of concentrated solar radiation for the synthesis of pyranopyrazole derivatives. This involved a solvent-free and catalyst-free approach employing a multi-component strategy. The aldehyde, ethyl acetoacetate, malononitrile, and hydrazine hydrate mixture was placed in a round-bottom flask and continuously stirred on a magnetic stirrer under concentrated solar radiation. The precipitate was observed within 3–4 min (Scheme 8). This method resulted in remarkable energy savings of approximately 98% compared to the conventional approach, while also exhibiting exceptional speed and high yields.

Despite having numerous advantages and significant environmental importance, the CSR method has not received extensive research attention. Therefore, efforts should be directed toward comprehending its characteristics to harness its complete potential.

### 2.3 Ultrasound irradiation technique

Ultrasonic waves induce cavitation, leading to the formation and collapse of bubbles, which create localized high temperatures and pressures, enhancing reaction rates. It reduces the need for elevated temperatures and potentially hazardous reagents while improving selectivity and purity due to milder reaction conditions.^42,43^

**Scheme 9:**
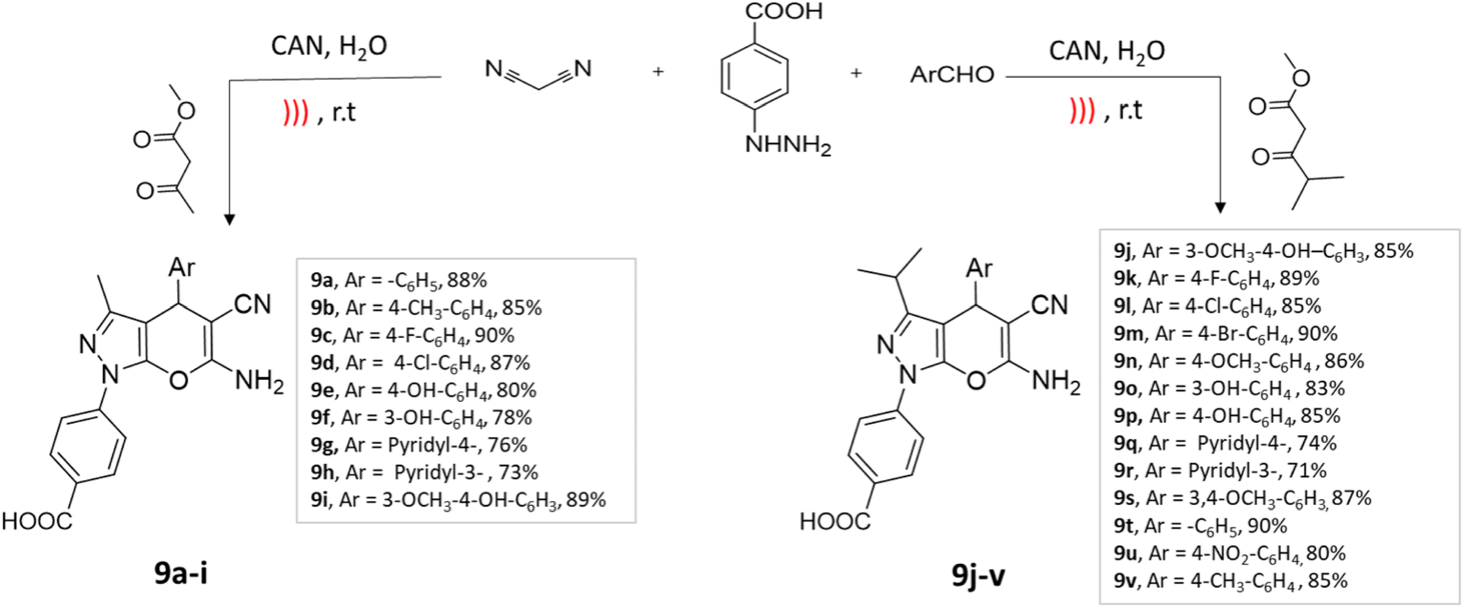
Synthesis of dihydropyrano[2,3-*c*]pyrazole derivatives using CAN under ultrasound-mediated technique.

An efficient four-component synthesis of dihydropyrano[2,3-*c*]pyrazole derivatives using ultrasound irradiation was reported by Ablajan *et al.*^44^ The desired compounds were successfully synthesized with favorable to exceptional yields using a ceric ammonium nitrate (CAN) catalyst in a water medium under the influence of ultrasound irradiation (Scheme 9).

**Scheme 10:**
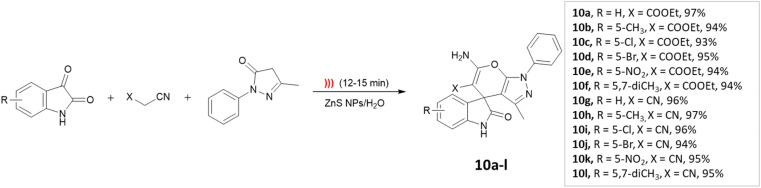
Synthesis of spiro[indoline-3,4′-pyrano[2,3-*c*]pyrazole] derivatives using ZnS NPs under ultrasonic radiation.

Dandia and coworkers^45^ utilized ZnS nanoparticles within a water environment under ultrasonic irradiation. The researchers conducted a one-pot three-component synthesis involving isatin, ethyl-cyanoacetate, and 3-methyl-1-phenyl-2-pyrazolin-5-one to successfully synthesize spiro[indoline-3,4′-pyrano[2,3-*c*]pyrazole] derivatives (Scheme 10). The catalyst could be reused for up to three cycles.

**Scheme 11:**
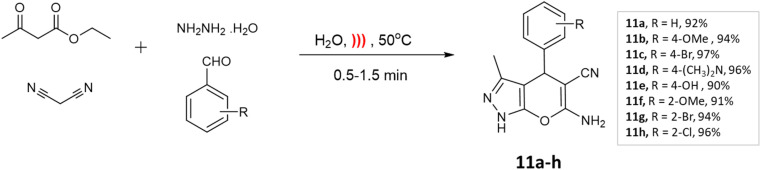
Synthesis of pyrano[2,3-*c*]pyrazoles in aqueous medium under ultrasonication.

Shabalala *et al.*^46^ reported the pyrano[2,3-*c*]pyrazole synthesis through a catalyst-free multicomponent reaction. This reaction involved ethyl acetoacetate, aromatic aldehydes, hydrazine monohydrate, and malononitrile in a water medium, facilitated by ultrasonic irradiation, yielding excellent results (Scheme 11).

**Scheme 12:**
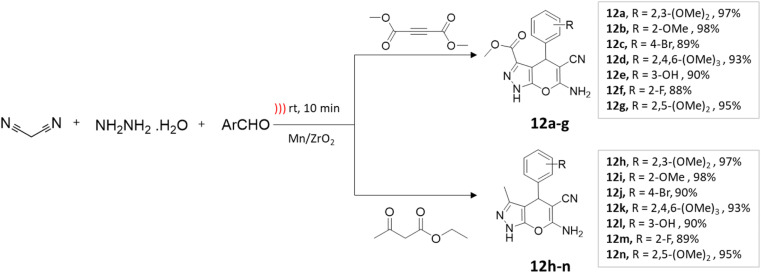
Synthesis of pyrano[2,3-*c*]pyrazole derivatives using Mn/ZrO_2_ under ultrasound-mediated technique.

Maddila and colleagues^47^ employed Mn/ZrO_2_ in the ultrasound-assisted synthesis of pyrano[2,3-*c*]pyrazole-3-carboxylate and pyrano[2,3-*c*]pyrazole-5-carbonitriles. This process entailed coupling reactions of ethyl acetoacetate or dimethylacetylenedicarboxylate, hydrazine hydrate, aromatic aldehyde, and malononitrile in an aqueous ethanol solution (Scheme 12). Under ultrasonication, a yield of 98% is obtained within 10 min, compared to an 83% yield achieved by conventional methods in 1 h (Table 2).

**Table 2:** A summary of ultrasound-assisted pyrano[2,3-*c*]pyrazole and spiropyrano[2,3-*c*] pyrazole synthesis

Product	Reactants	Catalyst	Solvent	Method employed	Reaction time	Yield	Ref.
Dihydropyrano[2,3-*c*]pyrazoles	Four-component system: 4-hydrazinobenzoic acid, β-keto esters, aromatic aldehydes, and malononitrile	Ceric ammonium nitrate (CAN)	Water	Ultrasound	45–60 min	70–90%	Ablajan *et al.*^44^
Spiro[indoline-3,4′-pyrano[2,3-*c*]]pyrazoles	Three-component system: isatin, ethyl-cyanoacetate, and 3-methyl-1-phenyl-2-pyrazolin-5-one	ZnS nanoparticles	Water	Ultrasound	12–15 min	93–97%	Dandia *et al.*^45^
Pyrano[2,3-*c*]pyrazoles	Four-component system: aromatic aldehydes, hydrazine monohydrate, ethyl acetoacetate, and malononitrile	Catalyst free	Water	Ultrasound	30–90 s	90–97%	Shabalala *et al.*^46^
Pyrano[2,3-*c*]pyrazole-3-carboxylate and pyrano[2,3-*c*]pyrazole-5-carbonitriles	Four-component system: dimethylacetylenedicarboxylate/ethyl acetoacetate, hydrazine hydrate, malononitrile, and aromatic aldehyde	Mn/ZrO_2_	Aq. ethanol	Ultrasound	10 min	88–99%	Maddila *et al.*^47^

For the Ultrasound-assisted reactions, we witness shortened reaction durations, with aqueous media commonly employed as the solvent, rendering these reactions environmentally friendlier in multiple aspects. However, there remains ample room to investigate the impact of Ultrasonic irradiation further in the organic synthesis of pyrano-pyrazoles, particularly concerning their medicinal and biological properties.

## 3 Catalysis in green processes

In organic synthesis, catalysis offers several advantages, including increased reaction rates, enhanced selectivity, and milder reaction conditions. It plays a pivotal role in reducing energy consumption, minimizing waste, and improving overall process efficiency. The key difference between conventional and green catalysis lies in their environmental impact. Conventional catalysts may involve toxic or costly materials, while green catalysts emphasize sustainability, utilizing biodegradable, renewable, or benign substances, resulting in more eco-friendly and efficient organic synthesis processes.^30,48,49^

### 3.1 Nano-catalysis

Nano-catalysis has evolved to provide rapid and sustainable routes, reducing waste and increasing reusability due to their high surface-to-volume ratio, allowing greener and more efficient synthesis of diverse heterocyclic structures.^50^ They often exhibit high selectivity, allowing for precise control over the desired reaction pathways, thereby minimizing the formation of unwanted byproducts. Furthermore, they are highly stable and durable, withstanding harsh reaction conditions and prolonged use without significant loss of activity.^16,51,52^

**Scheme 13:**
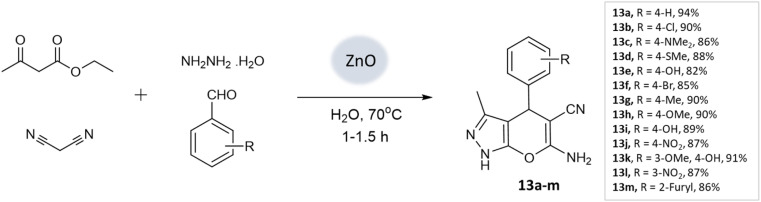
ZnO nanoparticle catalyzed one-pot four-component green synthesis of pyranopyrazoles.

S. U. Tekale and coworkers^53^ documented a method for synthesizing 4*H*-pyrano[2,3-*c*]pyrazoles, utilizing a zinc oxide nanoparticle-catalyzed multicomponent water-based reaction. The crystalline structure of the ZnO nanoparticles was confirmed through XRD investigations. Moreover, TEM analysis unveiled particle sizes spanning from 50 to 100 nm, creating an extensive surface area that facilitated the accelerated formation of the desired products. Employing an aqueous medium, a four-component coupling reaction involving ethyl acetoacetate, aromatic aldehyde, malononitrile, and hydrazine hydrate, along with ZnO nanoparticles as a catalyst, resulted in the production of pyranopyrazoles with elevated yields in a brief timeframe (Scheme 13).

**Scheme 14:**
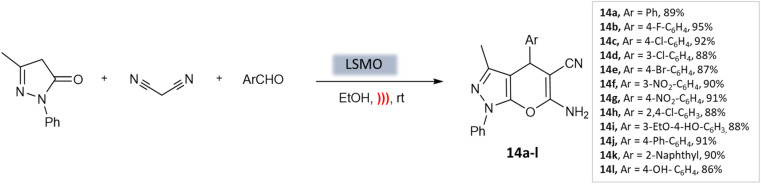
Nano-LSMO catalyzed synthesis of 4*H*-pyrano[2,3-*c*]pyrazole under ultrasonication.

Azarifar *et al.*^54^ developed highly efficient magnetic lanthanum strontium magnesium oxide (La_0.7_ Sr_0.3_MnO_3_ or LSMO) nanoparticles with remarkable swiftness. Magnetic nanoparticles (MNPs) are easily accessible, enabling their widespread use due to their stable catalyst linkages. Additionally, their simple separation using an external magnetic field streamlines the purification process. Moreover, they exhibit lower catalyst leaching compared to other material-supported catalysts, making them a highly promising choice for catalytic applications.^55,56^ The composite catalyst here, La_0.7_ Sr_0.3_MnO_3_, demonstrated outstanding characteristics such as a surface area of 39 m^2^ g^−1^, an average size of approximately 20 nm, and a magnetization of around 15 emu g^−1^. Using just 5 mol% of LSMO catalyst under ultrasound irradiation in an ethanol medium, researchers achieved high efficiency in producing pyrano-[2,3-*c*]-pyrazole scaffolds (Scheme 14). This process yielded excellent yields within 10 min at room temperature.

**Scheme 15:**
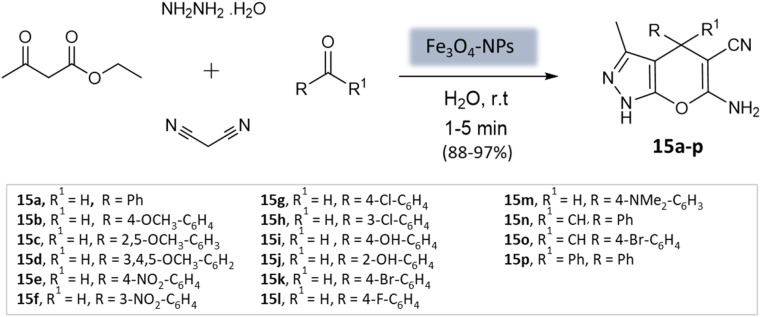
Magnetic Fe_3_O_4_ nanoparticles catalyzed four-component synthesis of pyranopyrazoles in aqueous medium.

El-Remaily *et al.*^57^ explored the use of magnetic Fe_3_O_4_ nanoparticles as a heterogeneous catalyst in the synthesis of pyranopyrazoles. This involved a four-component reaction, wherein a combination of ethyl acetoacetate, hydrazine hydrate, aldehydes or ketones, and malononitrile was reacted in a water medium at room temperature (Scheme 15). The best yields were obtained with 6 mol% Fe_3_O_4_-MNPs in aqueous media within 15 min. The catalyst could be reused up to fourteen times with no significant loss of catalytic activity.

**Scheme 16:**
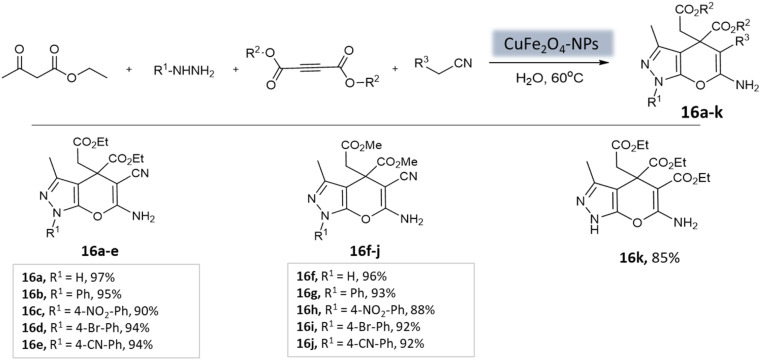
CuFe_2_O_4_ nanoparticles catalyzed synthesis of 3-methyl-1,4-dihydropyrano[2,3-*c*]pyrazole derivatives in aqueous medium.

Pradhan *et al.*^58^ successfully synthesized a highly efficient nanocatalyst called copper ferrite (CuFe_2_O_4_) using a straightforward citric acid complex method. This catalyst demonstrated effectiveness in synthesizing pyrano[2,3-*c*]-pyrazoles through a four-component reaction involving alkyl nitrile derivatives, various hydrazine derivatives, dialkyl acetylenedicarboxylate, and ethyl acetoacetate (Scheme 16). Notably, using just 8 mol% of CuFe_2_O_4_, the researchers achieved remarkable yields in water at 60 °C within 4 h. However, when the ethyl acetoacetate was replaced with dialkyl acetylenedicarboxylate, the desired product yields were unsatisfactory (12–43%).

**Scheme 17:**
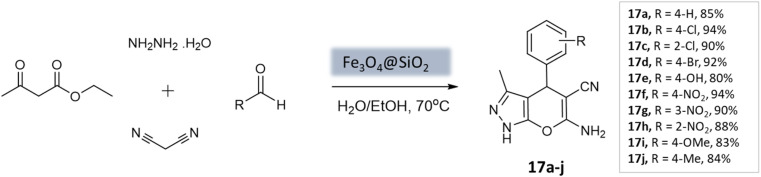
Fe_3_O_4_@SiO_2_ core–shell NPs. catalyzed synthesis of pyranopyrazole derivatives.

Soleimani *et al.*^59^ developed Fe_3_O_4_@SiO_2_ core–shell nanoparticles as a magnetically separable nanocatalyst for a four-component coupling reaction. This reaction involved the condensation of aromatic aldehydes, malononitrile, ethyl acetoacetate, and hydrazine hydrate in H_2_O/EtOH mixture to produce substituted pyranopyrazoles in high yields within 40 min (Scheme 17). The Fe_3_O_4_@SiO_2_ NPs had a roughly spherical morphology with some agglomeration. XRD analysis confirmed that the silica-coated iron oxide NPs retained the magnetic properties of the bare Fe_3_O_4_ NPs. The catalyst demonstrated durability and could be reused up to five times without significant loss in catalytic activity.

**Scheme 18:**
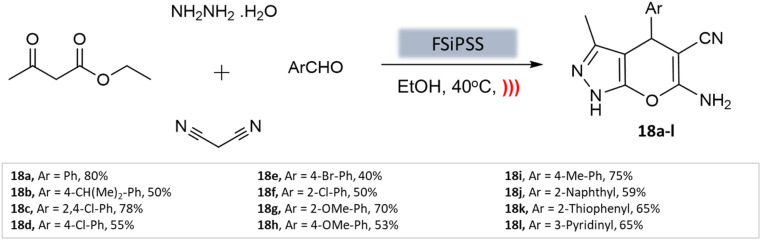
FSiPSS catalyzed synthesis of pyrano[2,3-*c*] pyrazole derivatives under ultrasonication.

A highly efficient and recoverable nanomagnetic catalyst, Fe_3_O_4_@SiO_2_@OSi(CH_2_)_3_–*N*(3-pyridoyl sulfonic acid) semicarbazide (FSiPSS), was designed, synthesized, and characterized using various techniques by Beiranvand *et al.*^60^ for the synthesis of diverse pyranopyrazole derivatives through a one-pot four-component condensation reaction of ethyl acetoacetate, hydrazine hydrate, aromatic aldehydes, malononitrile under ultrasonication (Scheme 18). The catalyst's specific surface area was found to be 35.6 m^2^ g^−1^ with an average size between 13.66 and 35.86 nm to facilitate the catalyst's effectiveness in carrying out the desired synthesis. The reaction achieved very short reaction times, good to high yields, and easy work-up. This novel nanomagnetic catalyst shows great potential for efficient and sustainable synthesis processes.

**Scheme 19:**
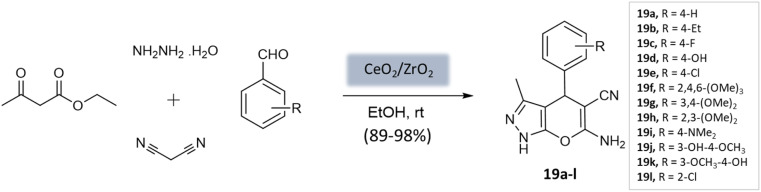
CeO_2_/ZrO_2_ catalyzed synthesis of pyranopyrazole derivatives at room temperature.

Maddila *et al.*^61^ introduced a ceria-doped zirconia catalyst prepared *via* the wet impregnation method for the synthesis of pyranopyrazoles with remarkable yields within 15 min at room temperature (Scheme 19). The four-component reaction, involving hydrazine hydrate, ethyl acetoacetate, malononitrile, and substituted aldehydes in ethanol, was efficiently catalyzed using CeO_2_/ZrO_2_. The catalyst could be easily recovered through filtration and recycled for up to six cycles while maintaining its efficiency.

**Scheme 20:**
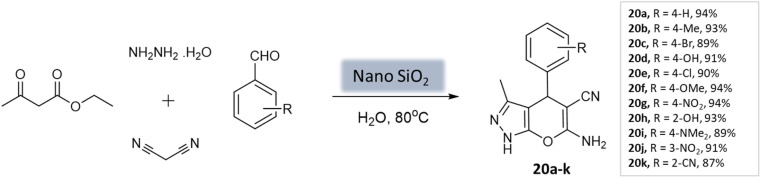
Nano SiO_2_ catalyzed synthesis of pyranopyrazole derivatives in aqueous medium.

Patel *et al.*^62^ utilized a recyclable nano-SiO_2_ catalyst to prepare pyrano[2,3-*c*]-pyrazoles. The catalyst was synthesized from wheat straw agricultural waste through the sol–gel process. The catalyst exhibited a uniform distribution and a spherical shape, with a crystallite size ranging from 100 to 200 nm. BET analysis revealed important properties, including a surface area of 215.6 m^2^ g^−1^, a pore volume of 0.269 cm^3^ g^−1^, and a pore diameter of 7.1 nm. The reaction involved hydrazine hydrate, malononitrile, aromatic aldehydes, and ethyl acetoacetate in water as a multi-component system. Notably, using only 10 mol% of the nanocatalyst yielded the best performance, achieving excellent yields within 40 s (Scheme 20). The catalyst remained stable for up to five runs without a significant decrease in activity.

**Scheme 21:**
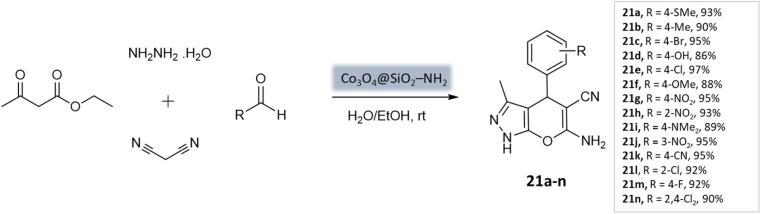
Co_3_O_4_@SiO_2_–NH_2_ catalyzed synthesis of pyranopyrazole derivatives at room temperature.

Shakiba Shahbazi *et al.*^63^ developed an efficient method to prepare dihydropyrano[2,3-*c*]pyrazoles *via* a multicomponent reaction of aryl aldehydes, malononitrile, ethyl acetoacetate, and hydrazine hydrate in the presence of SiO_2_@(3-aminopropyl)triethoxysilane-coated cobalt oxide (Co_3_O_4_) nanocomposite as the catalyst (Scheme 21). The nanocomposite had a cloudy and spherical shape, as observed in the FE-SEM image. Excellent yields and quick reaction times were obtained from the reaction, which can be attributed to the Bronsted–Lowry base's strong catalytic activity and high surface-to-volume ratio.

**Scheme 22:**
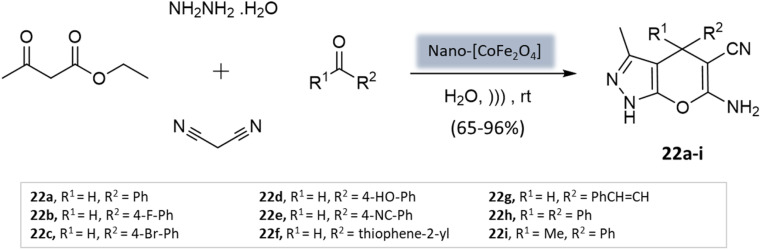
[CoFe_2_O_4_] NPs catalyzed synthesis of pyranopyrazole derivatives under ultrasonication.

Mishra *et al.*^64^ presented a novel method for synthesizing pyranopyrazole scaffolds employing nanomagnetic iron material as a reusable catalyst in an aqueous solvent under the ultrasonication technique. The protocol involved the condensation of malononitrile, hydrazine hydrate, ethyl acetoacetate, and substituted aldehydes with the CoFe_2_O_4_ catalyst (Scheme 22). Remarkably, both electron-withdrawing and electron-donating groups exhibited good reactivity and provided significant yields of the desired products.

**Scheme 23:**
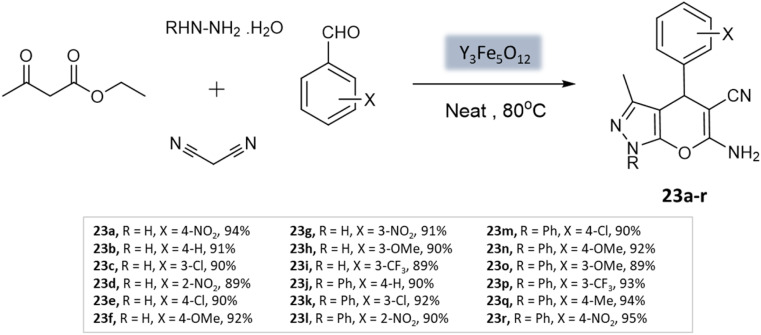
Y_3_Fe_5_O_12_ catalyzed synthesis of pyranopyrazole derivatives under solvent-free conditions.

Sedighinia *et al.*^65^ introduced a highly efficient and recyclable nanocatalyst called yttrium iron garnet (Y_3_Fe_5_O_12_; YIG). This catalyst was utilized for the synthesis of pyranopyrazoles through the combination of hydrazine hydrate, ethyl acetoacetate, malononitrile, and substituted aldehydes under solvent-free conditions at 80 °C. The reaction exhibited excellent yields within a short duration of 20 min (Scheme 23). The nanocatalyst could be easily recycled and maintained its activity for up to eight runs.

**Scheme 24:**
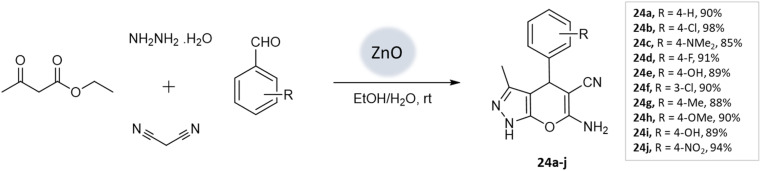
ZnO catalyzed synthesis of pyranopyrazole derivatives at room temperature.

Prakash Chhattise *et al.*^66^ used a hydrothermal technique to synthesize nanostructured ZnO. The catalytic activity of this nanostructured ZnO was evaluated as a heterogeneous catalyst in the multicomponent synthesis of pyranopyrazole derivatives (Scheme 24). XRD analysis confirmed the formation of highly crystalline ZnO with a wurtzite structure. FESEM analysis revealed the formation of submicron-sized spherical structures resembling marigold flowers. Remarkable yields were achieved within 15–30 min (Table 3).

**Table 3:** A summary of pyrano[2,3-*c*]pyrazoles and spiropyrano[2,3-*c*] pyrazoles synthesis using nanoparticle-catalysts

Product	Reactants	Catalyst	Solvent	Method employed	Reaction time	Yield	Ref.
4*H*-Pyrano[2,3-*c*]pyrazoles	Four-component system: aromatic aldehyde, malononitrile, ethyl acetoacetate, and hydrazine hydrate	Zinc oxide NPs	Water	Reflux, heating (70 °C)	1–1.5 h	82–94%	U. Tekale *et al.*^53^
Pyrano-[2,3-*c*]-pyrazoles	Three-component system: malononitrile, different aromatic aldehydes, and 3-methyl-pyrazolone	Lanthanum strontium magnesium oxide (MNPs)	Ethanol	Ultrasound	10 min	87–95%	Azarifar *et al.*^54^
Pyrano-[2,3-*c*]-pyrazoles	Four-component system: hydrazine hydrate, ethyl acetoacetate, aldehydes/ketones, and malononitrile	Magnetic Fe_3_O_4_ MNPs	Water	Room temperature stirring	1–5 min	88–97%	El-Remaily *et al.*^57^
Pyrano-[2,3-*c*]-pyrazoles	Four-component system: alkyl nitrile derivatives, various hydrazine derivatives, dialkyl acetylene dicarboxylate, and ethyl acetoacetate	Copper ferrite (CuFe_2_O_4_)	Water	Stirring (60 °C)	4 h	88–97%	Pradhan *et al.*^58^
Pyrano-[2,3-*c*]-pyrazoles	Four-component system: aromatic aldehydes, malononitrile, ethyl acetoacetate, and hydrazine hydrate	Fe_3_O_4_@SiO_2_ core–shell MNPs	Water/EtOH	Reflux, heating (70 °C)	40 min	80–94%	Soleimani *et al.*^59^
Pyrano-[2,3-*c*]-pyrazole derivatives	Four-component system: benzaldehydes, pyrazolones, and malononitriles	Fe_3_O_4_@SiO_2_@OSi(CH_2_)_3_–*N*(3-pyridoyl sulfonic acid) semicarbazide MNPs	EtOH	Ultrasound	3–7 min	40–80%	Beiranvand *et al.*^60^
Pyrano-[2,3-*c*]-pyrazoles	Four-component system: malononitrile, hydrazine hydrate, ethyl acetoacetate, and substituted aldehydes	Ceria-doped zirconia (CeO_2_/ZrO_2_) NPs	EtOH	Room temperature reflux	15 min	89–98%	Maddila *et al.*^61^
Pyrano-[2,3-*c*]-pyrazoles	Four-component system: hydrazine hydrate, malononitrile, aromatic aldehydes, and ethyl acetoacetate	SiO_2_ NPs	Water	Reflux, heating (80 °C)	40 min	87–94%	Patel *et al.*^62^
Dihydropyrano[2,3-*c*]pyrazoles	Four-component system: aryl aldehydes, malononitrile, ethyl acetoacetate, and hydrazine hydrate	SiO_2_@(3-aminopropyl)triethoxysilane-coated cobalt oxide (Co_3_O_4_) nanocomposite	Water/EtOH	Room temperature reflux	35–55 min	86–95%	Shahbazi *et al.*^63^
Pyrano-[2,3-*c*]-pyrazoles	Four-component system: malononitrile, hydrazine hydrate, ethyl acetoacetate, and substituted aldehydes	Nanomagnetic iron material [CoFe_2_O_4_]	Water	Ultrasound	5 min	65–96%	Mishra *et al.*^64^
Pyrano-[2,3-*c*]-pyrazoles	Four-component system: hydrazine hydrate, malononitrile, ethyl acetoacetate, and substituted aldehydes	Yttrium iron garnet (Y_3_Fe_5_O_12_; YIG)	Solvent free	Reflux, heating (80 °C)	20 min	89–95%	Sedighinia *et al.*^65^
Pyrano-[2,3-*c*]-pyrazoles	Four-component system: hydrazine hydrate, malononitrile, ethyl acetoacetate, and substituted aldehydes	Zinc oxide (ZnO) NPs	Water/EtOH	Room temperature reflux	15–30 min	85–98%	Chhattise *et al.*^66^

The eco-friendliness of nano-catalyst production relies on factors like selecting non-toxic materials and preparation techniques, impacting energy consumption.^67^ Recent research emphasizes creating safe, sustainable nano-catalysts through energy-efficient methods like microwave and ultrasound-assisted synthesis, solvent-free synthesis, template-directed synthesis, and more.^68^ Nevertheless, our primary focus is optimizing reaction parameters to increase pyranopyrazole yields using nano-catalysts tailored to specific applications, all within shorter reaction durations. Future investigations should prioritize cost-effective and environmentally benign metal/nanoparticle catalysts for pyranopyrazole synthesis, building upon the aforementioned references.

### 3.2 Organocatalysis

Organo-catalysis involves the use of small organic molecules as catalysts to facilitate chemical transformations. It's valuable in organic synthesis due to its compatibility with mild reaction conditions, often avoiding the need for transition metals. This approach offers advantages like atom economy, reduced environmental impact, and improved selectivity.^69,70^

**Scheme 25:**
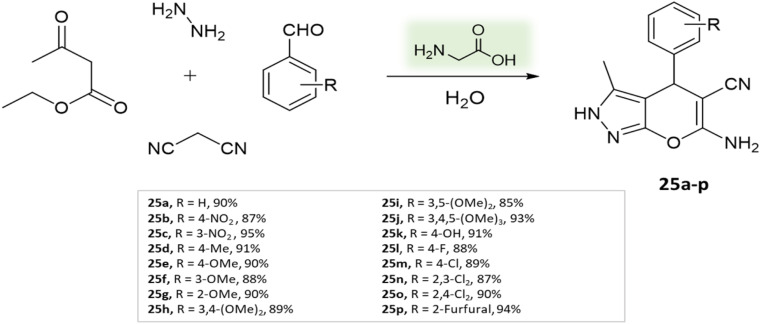
Synthesis of pyranopyrazole derivatives using Glycine.

Madhusudana Reddy and colleagues^71^ used an easily accessible, non-toxic, and environmentally friendly catalyst Glycine to synthesize pyranopyrazoles from ethyl acetoacetate, hydrazine hydrate, aldehyde, and malononitrile in aqueous medium at 25 °C in 5–20 min (Scheme 25).

**Scheme 26:**
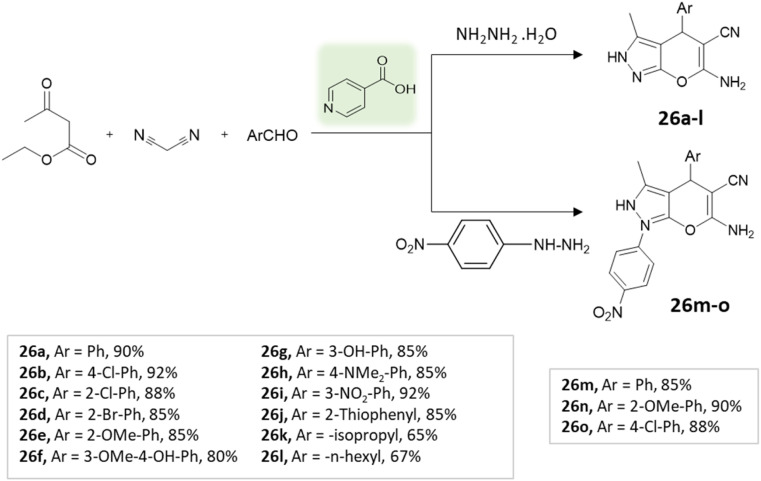
Synthesis of pyranopyrazole derivatives using isonicotinic acid.

Zolfigol *et al.* employed biological organocatalyst isonicotinic acid under solvent-free conditions^72^ to synthesize 1,4-dihydropyrano[2,3-*c*]pyrazoles through a four-component condensation reaction involving ethyl acetoacetate, malononitrile, aryl aldehydes, and hydrazine hydrate, carried out at a temperature of 85 °C (Scheme 26). Remarkably, the catalyst retained its catalytic efficacy within the boundaries of experimental error for four consecutive runs.

**Scheme 27:**
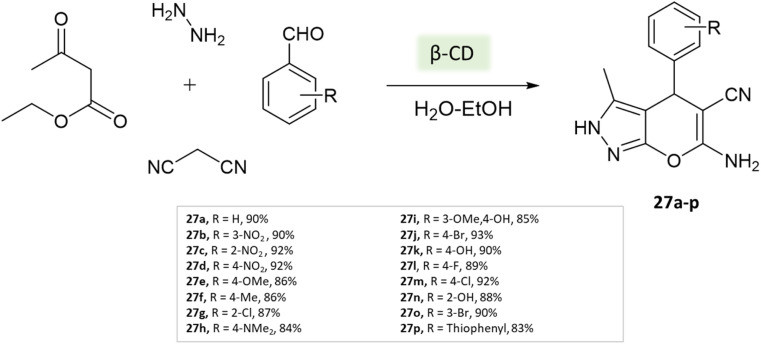
Synthesis of pyranopyrazole derivatives using β-cyclodextrin.

**Scheme 28:**
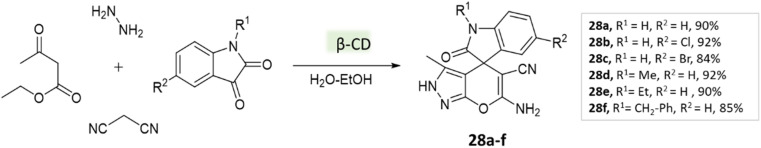
Synthesis of spiro-pyrano[2,3-*c*]pyrazole derivatives using β-cyclodextrin.

**Scheme 29:**
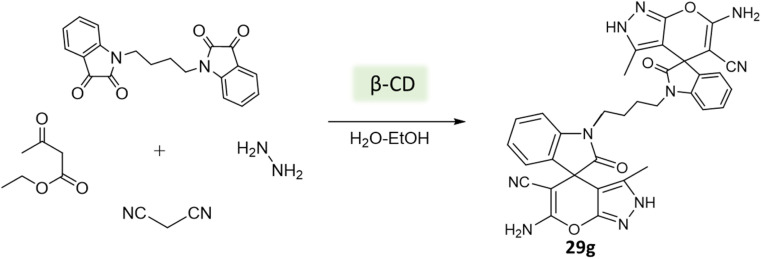
Synthesis of spiro-pyrano[2,3-*c*]pyrazole derivatives using β-cyclodextrin.

Tayade *et al.*^73^ utilized a biodegradable supramolecular β-cyclodextrin (β-CD) catalyst for the synthesis of pyrano[2,3-*c*]pyrazole and spiro-pyrano[2,3-*c*]pyrazole derivatives *via* MCR strategy. This process involved a reaction incorporating aldehydes, isatins, hydrazine hydrate, malononitrile, and β-ketoester, conducted in a mixture of H_2_O/EtOH at 80 °C (Schemes 27–29). Impressively, the catalyst retained its efficacy and could be reused for up to three cycles.

**Scheme 30:**
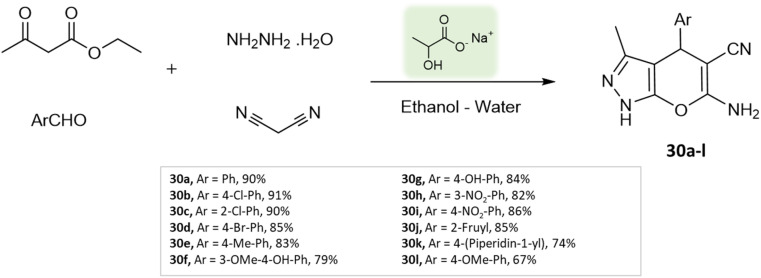
Synthesis of pyranopyrazole derivatives using sodium lactate.

J. P. Sonar *et al.*^74^ employed sodium lactate as a catalyst within an aqueous ethanolic environment under reflux conditions to synthesize pyranopyrazoles. This process involved the combination of hydrazine hydrate, ethyl acetoacetate, malononitrile, and various aldehydes (Scheme 30). The approach is characterized by its simplicity and environmentally friendly nature, leading to the production of pyranopyrazoles with moderate to excellent yields in a short span of reaction time (Table 4).

**Table 4:** A summary of pyrano[2,3-*c*]pyrazoles and spiropyrano[2,3-*c*] pyrazoles synthesis using organo-catalysts

Product	Reactants	Catalyst	Solvent	Method employed	Reaction time	Yield	Ref.
Pyrano-[2,3-*c*]-pyrazoles	Four-component system: ethyl acetoacetate, hydrazine hydrate, an aldehyde, and malononitrile	Glycine	Water	Room temperature stirring	5–20 min	85–94%	M. Reddy *et al.*^71^
1,4-Dihydropyrano[2,3-*c*]pyrazoles	Four-component system: malononitrile, hydrazine hydrate, ethyl acetoacetate, and aryl aldehydes	Isonicotinic acid	Solvent free	Reflux, heating (85 °C)	10–15 min	65–92%	Zolfigol *et al.*^72^
Pyrano[2,3-*c*]pyrazole and spiro-pyrano[2,3-*c*]pyrazole derivatives	Four-component system: aldehydes, isatins, hydrazine hydrate, malononitrile, and β-ketoester	β-Cyclodextrin (β-CD)	Water/EtOH	Reflux, heating (80 °C)	15–50 min	83–93%	Tayade *et al.*^73^
Pyrano-[2,3-*c*]-pyrazoles	Four-component system: hydrazine hydrate, malononitrile, ethyl acetoacetate, and substituted aldehydes	Sodium lactate	Water/EtOH	Room temperature reflux	10–20 min	67–91%	J. P. Sonar *et al.*^74^

Organo-catalysis stands out as the safest and most efficient method to synthesize pyranopyrazoles offering mild reaction conditions with impressive yields. It can be done in aqueous media, thus, eliminating the need for toxic solvents and reducing conventional reaction time. This eco-friendly approach warrants further exploration in advancing pyranopyrazole synthesis.

### 3.3 Bio-catalysis/natural catalysis

Bio-catalysis involves the use of natural catalysts like enzymes to drive chemical reactions and has gained prominence due to its specificity and compatibility with mild conditions, minimizing byproducts and waste. In organic synthesis, it offers regio- and stereoselectivity, enabling complex transformations.^75^

**Scheme 31:**
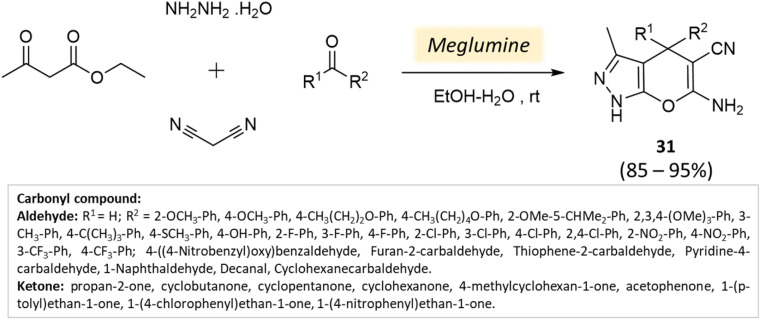
Synthesis of pyranopyrazole derivatives using meglumine.

**Scheme 32:**
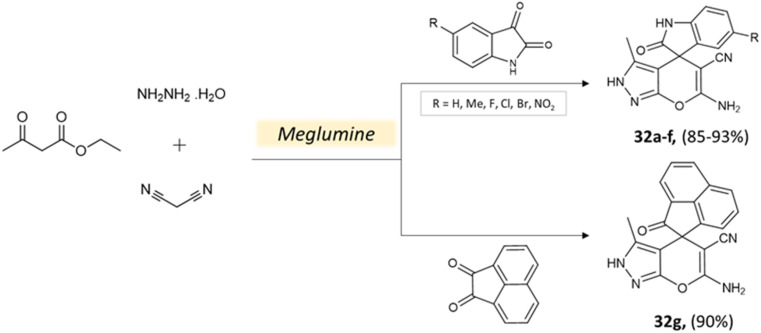
Synthesis of spiro[indoline-pyrano[2,3-*c*]-pyrazole] derivatives using meglumine.

Guo *et al.*^76^ used a bio-based chemical catalyst meglumine to develop a series of pyranopyrazoles and spiro[indoline-pyrano[2,3-*c*]-pyrazole] derivatives. A four-component reaction scheme was employed involving malononitrile, hydrazine hydrate, β-keto ester, and carbonyl compound or isatin in a solvent mixture of EtOH–H_2_O at room temperature (Schemes 31 and 32). The reaction, facilitated by 10 mol% meglumine, achieved excellent yields. The catalyst demonstrated reusability for up to 3 cycles with minimal loss of activity.

**Scheme 33:**
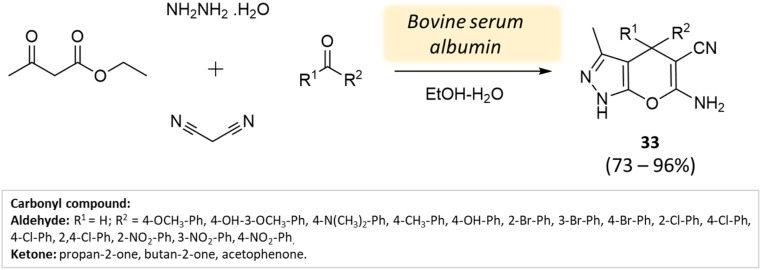
Synthesis of pyrano[2,3-*c*]-pyrazole derivatives using bovine serum albumin.

**Scheme 34:**
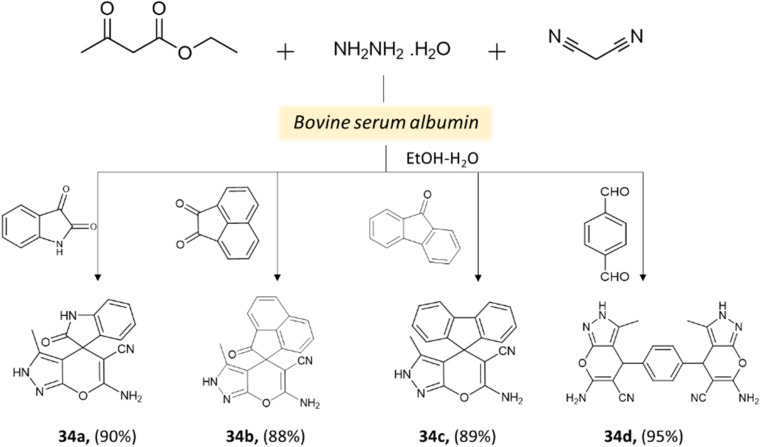
Synthesis of pyrano[2,3-*c*]-pyrazole derivatives using bovine serum albumin.

Xingtian and colleagues^77^ synthesized bovine serum albumin (BSA) and utilized it as a catalyst for generating pyrano[2,3-*c*]-pyrazole derivatives and spiro[indoline-pyrano[2,3-*c*]-pyrazole] derivatives. The catalytic performance of BSA was evaluated in a four-component reaction involving hydrazine hydrate, malononitrile, ethyl acetoacetate, and various carbonyl compounds. The reaction took place in an ethanol system at 45 °C for 45 min, resulting in excellent yields (Schemes 33 and 34). The recovered BSA could be reused for up to five cycles with alike effectiveness.

**Scheme 35:**
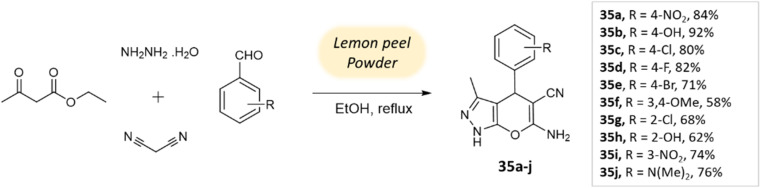
Synthesis of pyrano[2,3-*c*]-pyrazole derivatives using lemon peel powder.

Ghodke *et al.*^78^ developed a facile method for synthesizing pyrano[2,3-*c*]pyrazoles *via* a component condensation reaction with lemon peel powder serving as a natural catalyst. In this process, malononitrile, aldehydes, ethyl acetoacetate, and hydrazine hydrate are reacted in ethanol under reflux conditions, with lemon peel powder (Scheme 35).

**Scheme 36:**
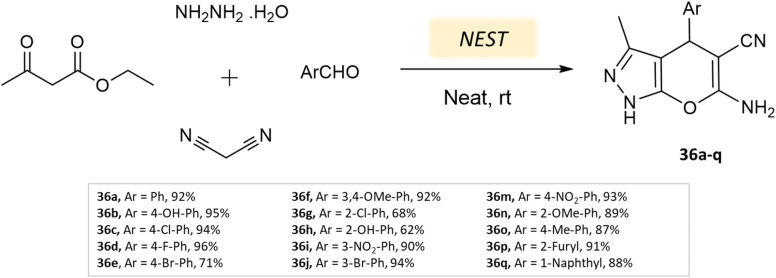
Synthesis of pyrano[2,3-*c*]-pyrazole derivatives using nano-eggshell/Ti(iv) as a catalyst.

Arefeh Dehghani *et al.*^79^ effectively synthesized dihydropyrano[2,3-*c*]pyrazoles using nano-eggshell/Ti(iv) as a catalyst through a four-component reaction comprising ethyl acetoacetate, hydrazine hydrate, malononitrile, and aldehydes at room temperature under solvent-free conditions (Scheme 36). The technique offers notable advantages such as mild reaction conditions, quick reaction times, simple purification processes, excellent yields, the potential for catalyst reusability, and the removal of harmful organic solvents.

**Scheme 37:**
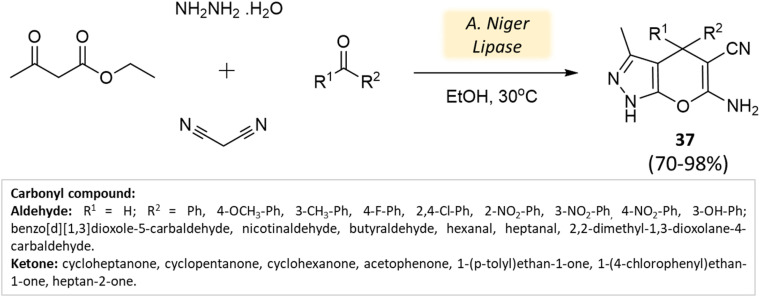
Synthesis of pyrano[2,3-*c*]-pyrazole derivatives using *A. niger* lipase.

Bora and coworkers^80^ showcased the dihydropyran-[2,3-*c*]-pyrazole synthesis utilizing *Aspergillus niger* lipase as a catalyst. This lipase enzyme adeptly facilitated a four-component condensation reaction involving ethyl acetoacetate, hydrazine hydrate, malononitrile, and either aldehyde or ketone. Remarkable yields were achieved at a temperature of 30 °C (Scheme 37). Furthermore, the biocatalyst demonstrated reusability for up to three cycles.

**Scheme 38:**
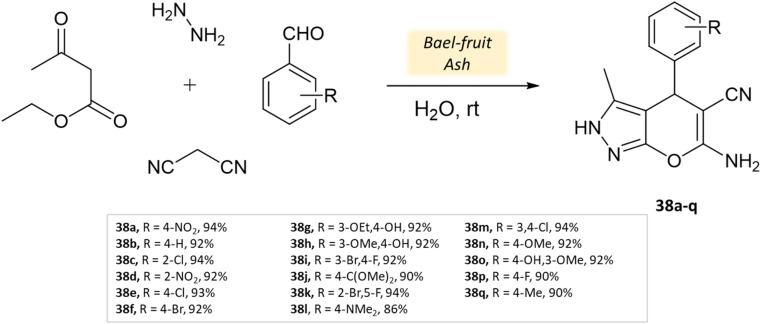
Synthesis of pyrano[2,3-*c*]-pyrazole derivatives using Bael fruit ash.

Shinde and his research team^81^ successfully developed a synthesis method using a natural catalyst made of bael fruit ash. Its efficacy was evaluated in synthesizing pyrano[2,3-*c*]-pyrazoles and pyrazolyl-4*H*-chromene derivatives in an aqueous medium through four-component reactions involving ethyl acetoacetate, malononitrile, hydrazine hydrate, various aldehydes. These reactions were conducted at room temperature for a duration of 30 min (Scheme 38). The method was notably suitable for producing pyrazolyl-4*H*-chromenes, yielding excellent results ranging from 86% to 94% for different salicylaldehydes (Table 5). Impressively, the BFA catalyst demonstrated stability across five cycles, exhibiting minimal loss of activity.

**Table 5:** A summary of pyrano[2,3-*c*]pyrazoles and spiropyrano[2,3-*c*] pyrazoles synthesis using bio-catalysts

Product	Reactants	Catalyst	Solvent	Method employed	Reaction Time	Yield	Ref.
Spiro[indoline-pyrano[2,3-*c*]-pyrazole] derivatives	Four-component system: malononitrile, hydrazine hydrate, β-keto ester, and carbonyl compound or isatin	Meglumine	Water/EtOH	Room temperature stirring	25–40 min	85–95%	Guo *et al.*^76^
Pyrano[2,3-*c*]-pyrazole and spiro[indoline-pyrano[2,3-*c*]-pyrazole] derivatives	Four-component system: hydrazine hydrate, malononitrile, ethyl acetoacetate, and various carbonyl compounds	Bovine serum albumin (BSA)	Water/EtOH	Reflux, heating (45 °C)	45 min	73–96%	Xingtian *et al.*^77^
Pyrano-[2,3-*c*]-pyrazoles	Four-component system: aldehydes, malononitrile, ethyl acetoacetate, and hydrazine hydrate	Lemon peel powder	Ethanol	Room temperature reflux	2–5 h	58–92%	Ghodke *et al.*^78^
Dihydropyrano[2,3-*c*]pyrazoles	Four-component system: aldehydes, malononitrile, ethyl acetoacetate, and hydrazine hydrate	Nano-eggshell/Ti(iv)	Solvent free	Room temperature stirring	8–25 min	62–96%	Dehghani *et al.*^79^
Dihydropyrano[2,3-*c*]pyrazoles	Four-component system: aldehydes, malononitrile, ethyl acetoacetate, and hydrazine hydrate	*Aspergillus niger* lipase (ANL)	Ethanol	Vigorous stirring, heating (30 °C)	60 min	70–98%	Bora *et al.*^80^
Pyrano[2,3-*c*]-pyrazoles and pyrazolyl4*H*-chromene derivatives	Four-component system: aldehydes/salicaldehydes, malononitrile, ethyl acetoacetate, and hydrazine hydrate	Bael fruit ash (BFA)	Water	Room temperature stirring	30 min	86–94%	Shinde *et al.*^81^

Bio-catalysis is an entirely eco-friendly method that eliminates reliance on metal catalysts and emphasizes natural products. Yet, further research is necessary to enhance reaction speed through a comprehensive understanding of catalytic efficiency and mechanisms.

## 4 Green solvents

Green solvents are crucial in organic synthesis due to their reduced environmental impact and health hazards compared to conventional solvents. Their use aligns with eco-friendly principles, driving cleaner and more sustainable organic synthetic practices.^82^ For instance, water, supercritical CO_2_, and ionic liquids have gained prominence. They offer advantages like improved atom economy, lower toxicity, and easier product separation.^83^

### 4.1 Water

Water has emerged as a prominent green solvent in organic synthesis due to its abundance, low cost, and environmental benignity. Water's characteristics, including its high polarity and unique hydrogen bonding, facilitate various reactions. Advantages include improved safety, minimized waste, and facile product isolation. It promotes eco-friendly synthesis for heterocyclic compounds such as pyrano-pyrazoles by enabling milder conditions and reducing the need for toxic organic solvents, thereby aligning with green chemistry principles.^84,85^

**Scheme 39:**
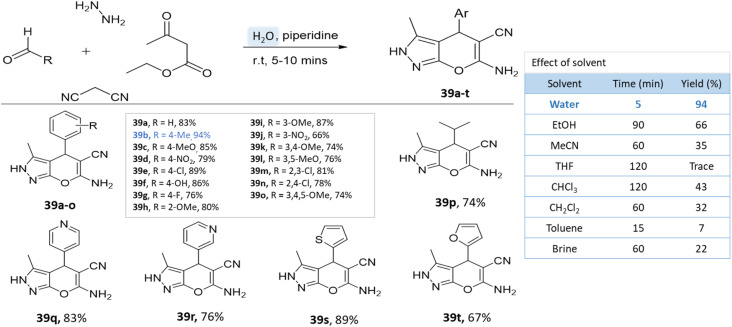
Synthesis of pyranopyrazole derivatives in aqueous medium using piperidine.

A series of pyranopyrazole derivatives were synthesized in an aqueous medium by Vasuki and Kumaravel^86^ through the multi-component strategy. At room temperature, the reaction was carried out between ethyl acetoacetate, hydrazine hydrate, malononitrile, and various benzaldehyde in the presence of piperidine. Water proved to be the most effective solvent when compared to typical organic solvents (Scheme 39).

**Scheme 40:**
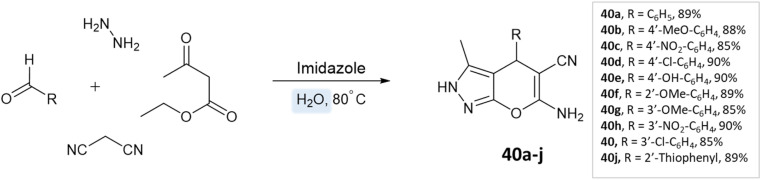
Synthesis of pyrano[2,3-*c*]pyrazole derivatives in aqueous medium using imidazole.

Siddekha *et al.*^87^ investigated the influence of different solvents dichloromethane (DCM), acetonitrile (CH_3_CN), ethanol, and water, for a model reaction of pyrano[2,3-*c*]pyrazole synthesis, employing a small amount of imidazole as an organocatalyst. Among the solvents tested, water demonstrated the highest yields within a relatively short reaction time of 20–30 min. Consequently, the aromatic aldehydes, malononitrile, hydrazine hydrate, ethyl acetoacetate, and imidazole were dissolved in water and the reaction mixture was heated on a preheated hot plate at 80 °C for 20–30 min (Scheme 40).

**Scheme 41:**
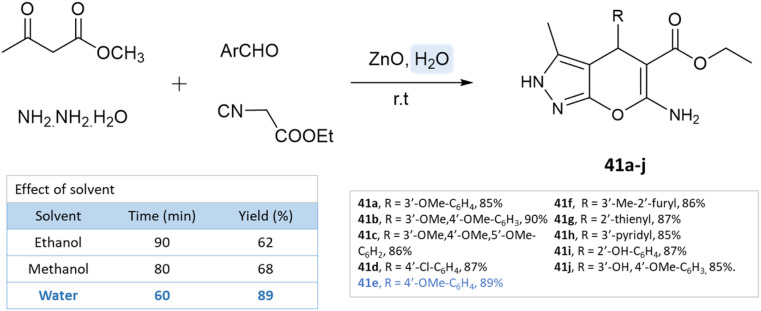
Pyrano[2,3-*c*]pyrazole-5-carboxylate synthesis in an aqueous medium using ZnO NPs.

Using ZnO nanoparticles at room temperature, Sachdeva and Saroj^88^ investigated the solvent effect for the synthesis of pyrano[2,3-*c*]pyrazole-5-carboxylate derivatives. While yields in ethanol and methanol are poor, the best results are obtained with water (Scheme 41).

**Scheme 42:**
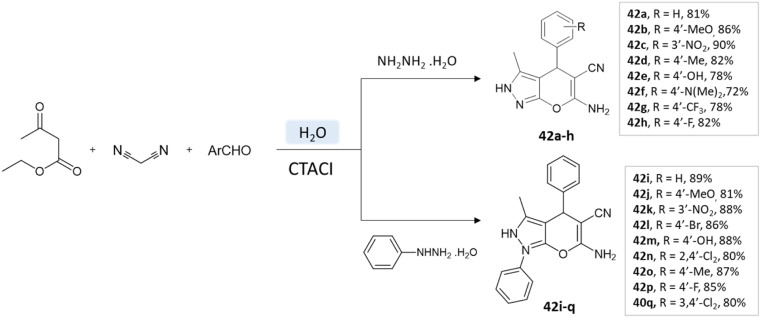
Pyrano[2,3-*c*] pyrazole synthesis in an aqueous medium using cetyltrimethyl-ammonium chloride.

A study conducted by Mingshu Wu *et al.*^89^ presented a facile approach for the 6-amino-3-methyl-4-aryl-(1-phenyl)-1,4-dihydropyrano[2,3-*c*]pyrazole-5-carbonitrile synthesis, utilizing water as the solvent. An aqueous solution of hydrazine hydrate or phenylhydrazine with ethyl acetoacetate was added with CTACl (cetyl-trimethyl-ammonium chloride), malononitrile, and substituted aldehyde (Scheme 42).

**Scheme 43:**
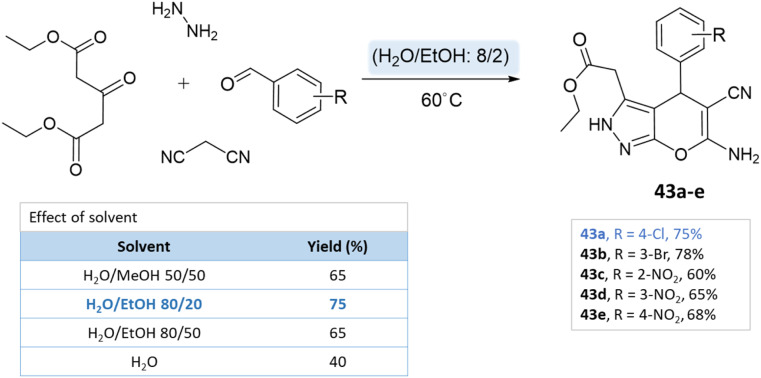
Pyrano[2,3-*c*] pyrazole synthesis in water/ethanol medium.

**Scheme 44:**
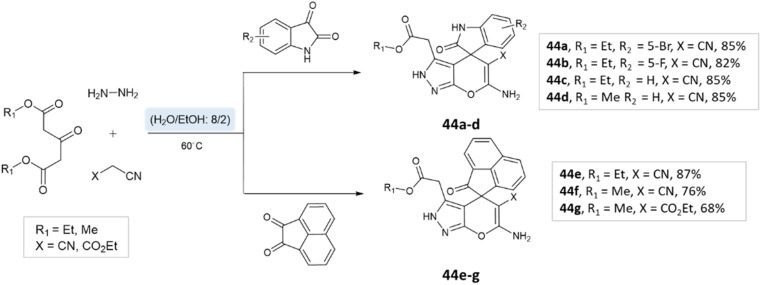
Spiro-pyrano[2,3-*c*] pyrazole synthesis in water/ethanol medium.

Majid Koohshari *et al.*^90^ have outlined a method for synthesizing pyrano[2,3-*c*]pyrazoles where dialkyl 3-oxopentanedioate, aromatic aldehydes containing electron-withdrawing groups were taken, along with hydrazine hydrate and malononitrile. The reaction takes place in a water/ethanol mixture without the need for a catalyst (Scheme 43). This method was effectively used to produce the spiro-pyrano[2,3-*c*] pyrazoles from carbonyl compounds, isatin derivatives, and acenaphthenequinone (Scheme 44).

**Scheme 45:**
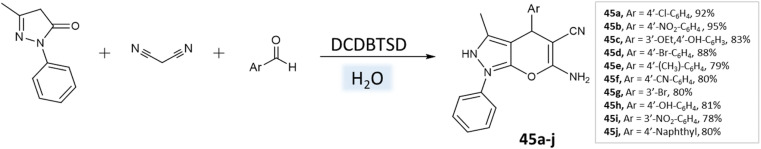
Synthesis of pyrano[2,3-*c*]pyrazole derivatives in water using DCDBTSD.

**Scheme 46:**
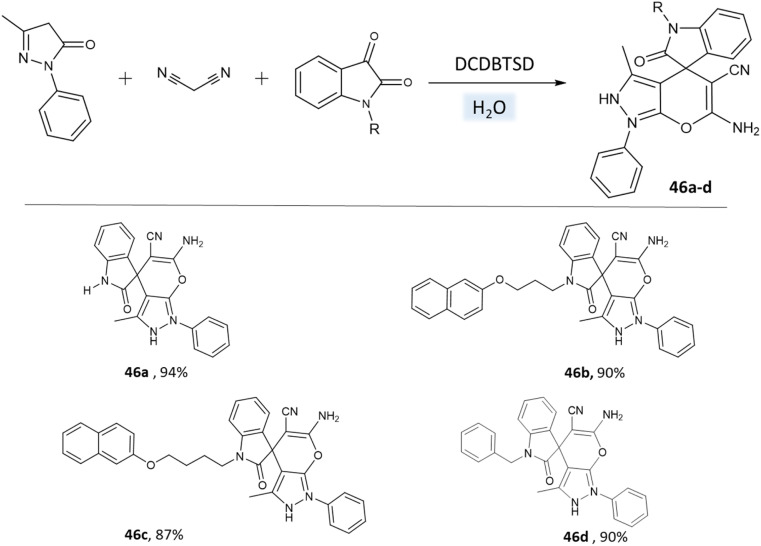
Synthesis of spiro-pyrano[2,3-*c*]pyrazole derivatives in water using DCDBTSD.

Khazaei *et al.*^91^ employed DCDBTSD (*N*,2-dibromo-6-chloro-3,4-dihydro-2*H*-benzo[1,2,4]thiadiazine-7-sulfonamide-1,1-dioxide) catalyst to facilitate the synthesis of 1,4-dihydropyrano[2,3-*c*]pyrazoles and spiro-pyrano[2,3-*c*]pyrazoles in an aqueous medium *via* multicomponent reaction (Schemes 45 and 46). This method was also applied for the preparation of 4*H*-pyran, pyrazolo[1,2-*b*]phthalazine, and spirooxindoles, as detailed in the report.

**Scheme 47:**
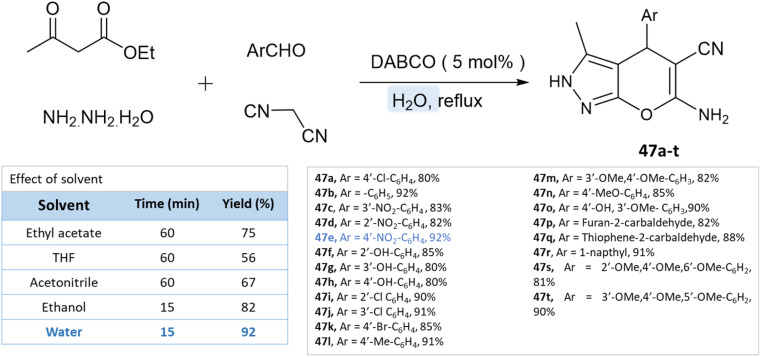
Synthesis of pyrano[2,3-*c*]pyrazole derivatives in aqueous medium using DABCO.

As reported by Waghmare *et al.*^92^ dihydropyrano[2,3-*c*]pyrazoles were synthesized in an aqueous medium with DABCO (1,4-diazabicyclo[2.2.2]octane) *via* a four-component reaction of ethylacetoacetate, hydrazine hydrate, malononitrile, and various aldehydes. Various solvents were studied for the probe reaction, including ethyl acetoacetate, tetrahydrofuran, acetonitrile, ethanol, and water. Water produced the highest yield when compared to organic solvents (Scheme 47).

**Scheme 48:**
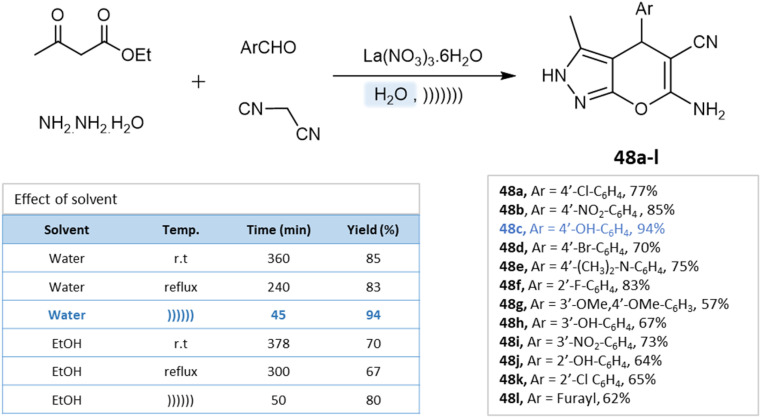
Synthesis of pyrano[2,3-*c*]pyrazole derivatives in aqueous medium using lanthanum(iii) nitrate.

Under the ultrasound irradiation method, Priya M. Khandare *et al.*^93^ synthesized pyranopyrazoles in an aqueous medium using lanthanum(iii) nitrate as a catalyst. The reaction conditions were optimized by performing the model reaction of 4-hydroxybenzaldehyde, ethyl acetoacetate, hydrazine hydrate, and lanthanum(iii) nitrate under different conditions. Using the ultrasonication method and water as a solvent, high yields were achieved in a short time (Scheme 48).

**Scheme 49:**
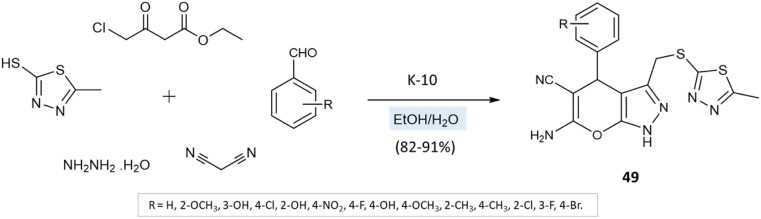
Synthesis of thiadiazole-pyranopyrazole derivatives in water/ethanol medium using K-10 clay.

A series of thiadiazole-pyranopyrazole derivatives was amalgamated by M. Reddy *et al.*^94^*via* the multicomponent reaction of 5-methyl-1,3,4-thiadiazole-2-thiol, hydrazine hydrate, ethyl 4-chloro-3-oxobutanoate, malononitrile, and aryl aldehydes using K10 clay as a green catalyst and ethanol–water as solvent media (Scheme 49). Furthermore, the reaction was also conducted in solvent-free media, leading to low yields with impurities. Solvent-mediated reactions, however, resulted in quantitative yields with no byproducts or impurities.

**Scheme 50:**
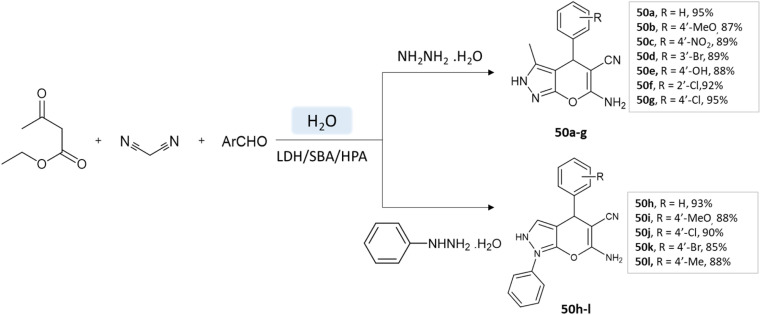
Synthesis of pyrano[2,3-*c*]pyrazole derivatives in aqueous medium using LDH/SBA/HPA.

**Scheme 51:**
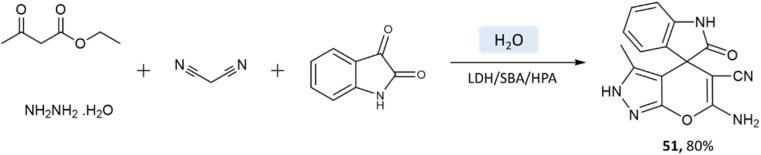
Synthesis of spiro-pyrano[2,3-*c*]pyrazole derivatives in water using LDH/SBA/HPA.

Samahe Sadjadi *et al.*^95^ examined the catalytic activity of a ternary hybrid catalyst including HPA (heteropolyacids), LDH (layered double hydroxides), and SBA-15 (mesoporous silica) for the development of pyranopyrazole and spiro-pyranopyrazoles derivatives in aqueous media. A mixture of hydrazine hydrate or phenylhydrazine, ethyl acetoacetate, malononitrile, and aldehyde in the presence of LDH/SBA/HPA was refluxed in water for 15 min (Schemes 50 and 51).

**Scheme 52:**
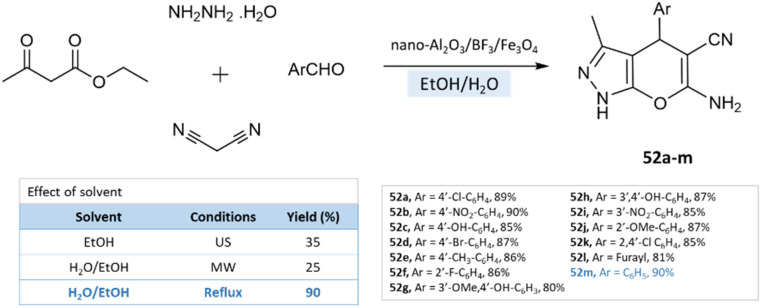
Synthesis of pyrano[2,3-*c*]pyrazole derivatives in the water–ethanol medium using nano-Al_2_O_3_/BF_3_/Fe_3_O_4_.

Under reflux conditions with water/ethanol, dihydropyrano[2,3-*c*]pyrazoles were synthesized from hydrazine hydrate, ethyl acetoacetate, malononitrile, and benzaldehyde by Babaei and Mirjalili^96^ using nano-Al_2_O_3_/BF_3_/Fe_3_O_4_ as catalysts (Scheme 52).

**Scheme 53:**
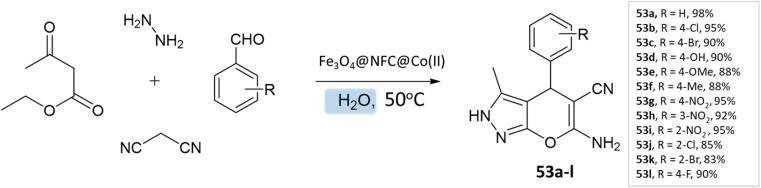
Synthesis of pyrano[2,3-*c*]pyrazole derivatives in aqueous medium using Fe_3_O_4_@NFC@Co(ii) as the catalyst.

G. Kargar and coworkers^97^ synthesized pyranopyrazole derivatives in an aqueous medium using a multi-core catalyst Fe_3_O_4_@NFC@Co(ii) from ethylacetoacetate, hydrazine hydrate, aldehyde, malononitrile, and Fe_3_O_4_@NFC@Co(ii) with vigorous stirring at 50 °C, resulting in excellent yields of the pyranopyrazoles in a short time (Scheme 53).

**Scheme 54:**
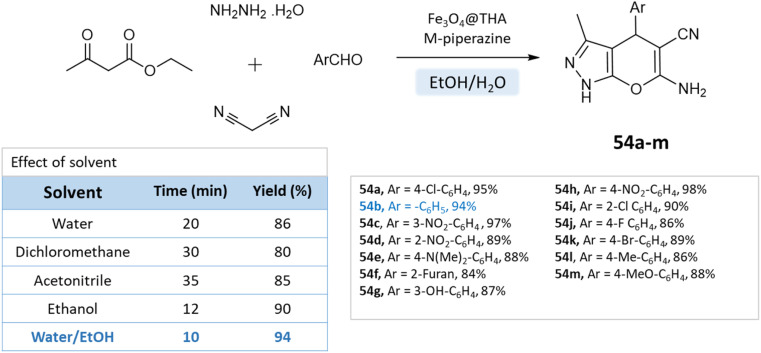
Synthesis of pyrano[2,3-*c*]pyrazole derivatives in water–ethanol medium using Fe_3_O_4_@THAM-piperazine as the catalyst.

Fatemeh Mir *et al.*^98^ reported the synthesis of dihydropyrano[2,3-*c*]pyrazole derivatives using a reusable Fe_3_O_4_@THAM-piperazine catalyst. The reaction is carried out in an ethanol/water medium, where initially 3-methyl-2-pyrazoline-5-one was precipitated using the reaction between hydrazine hydrate, and ethyl acetoacetate at room temperature, to which then aromatic aldehydes, malononitrile, Fe_3_O_4_@THAM-piperazine were added to the reaction mixture and stirred for the suitable time at 60 °C (Scheme 54).

Given its safety, lack of toxicity, and natural abundance, water as a solvent offers a promising direction for pyrano-pyrazole synthesis. Nonetheless, careful selection of reaction parameters, including temperature, time, and catalyst, is crucial to optimize the yields (Table 6). Variability in mechanisms must be considered and managed accordingly.

**Table 6:** A summary of pyrano[2,3-*c*]pyrazole and spiropyrano[2,3-*c*] pyrazole synthesis using water

Product	Reactants	Catalyst	Solvent	Method employed	Reaction time	Yield	Ref.
Pyrano-[2,3-*c*]-pyrazoles	Four-component system: hydrazine hydrate, ethyl acetoacetate, malononitrile, and various benzaldehydes	Piperidine	Water	Room temperature reflux	5–10 min	66–94%	G. Vasuki and Kumaravel^86^
Pyrano-[2,3-*c*]-pyrazoles	Four-component system: aromatic aldehydes, malononitrile, ethyl acetoacetate, hydrazine hydrate	Imidazole	Water	Reflux, heating (80 °C)	20–30 min	85–90%	Siddekha *et al.*^87^
Pyrano[2,3-*c*]pyrazole-5-carboxylate derivatives	Four-component system: aromatic aldehyde, ethyl 3-cyanopropanoate, ethyl acetoacetate, hydrazine hydrate	ZnO NPs	Water	Room temperature stirring	30–60 min	85–90%	Sachdeva and Saroj^88^
6-Amino-3-methyl-4-aryl-(1-phenyl)-1,4-dihydropyrano[2,3-*c*]pyrazole-5-carbonitrile	Four-component system: aldehyde, malononitrile, phenylhydrazine or hydrazine hydrate, and ethyl acetoacetate	CTACl	Water	Reflux, heating (90 °C)	4 h	72–90%	Mingshu Wu *et al.*^89^
Pyrano[2,3-*c*]pyrazoles	Four-component system: dialkyl 3-oxopentanedioate, aromatic aldehydes, hydrazine, and malononitrile	Catalyst free	Water/EtOH	Reflux, heating (60 °C)	12 h	60–78%	Koohshari *et al.*^90^
1,4-Dihydropyrano[2,3-*c*]pyrazoles and spiro-pyrano[2,3-*c*]pyrazoles	Four-component system: aromatic aldehydes, malononitrile, ethyl acetoacetate, hydrazine hydrate	DCDBTSD	Water	Reflux, heating (80 °C)	20–45 min	79–95%	Khazaei *et al.*^91^
Dihydropyrano[2,3-*c*]pyrazoles	Four-component system: ethylacetoacetate, hydrazine hydrate, malononitrile and various aldehydes	DABCO	Water	Room temperature reflux	15 min	80–92%	Waghmare *et al.*^92^
Pyrano[2,3-*c*]pyrazoles	Four-component system: 4-hydroxybenzaldehyde, ethyl acetoacetate, hydrazine hydrate, malononitrile	Lanthanum(iii) nitrate	Water	Ultrasound	45–60 min	62–94%	M. Khandare *et al.*^93^
Thiadiazole-pyranopyrazoles	Four-component system: 5-methyl-1,3,4-thiadiazole-2-thiol, ethyl 4-chloro-3-oxo butanoate, hydrazine hydrate, malononitrile, and aryl aldehydes	K10 clay	Water/EtOH	Heating (65–70 °C)	5 h	82–91%	M. Reddy *et al.*^94^
Pyrano[2,3-*c*]pyrazole and spiro-pyrano[2,3-*c*]pyrazoles	Four-component system: hydrazine hydrate or phenylhydrazine, ethyl acetoacetate, aldehyde, and malononitrile	Ternary hybrid catalyst including HPA, LDH, and SBA-15	Water	Reflux	15 min	85–95%	Samahe Sadjadi *et al.*^95^
Dihydropyrano[2,3-*c*]pyrazoles	Four-component system: hydrazine hydrate, ethyl acetoacetate, malononitrile, and benzaldehyde	Nano-Al_2_O_3_/BF_3_/Fe_3_O_4_	Water/EtOH	Reflux	25–40 min	80–90%	Babaei and Mirjalili^96^
Pyrano[2,3-*c*]pyrazoles	Four-component system: hydrazine hydrate, ethyl acetoacetate, aldehyde, malononitrile	Fe_3_O_4_@NFC@Co(ii)	Water	Reflux, heating (50 °C)	10–20 min	83–98%	G. Kargar *et al.*^97^
Pyrano[2,3-*c*]pyrazoles	Four-component system: hydrazine hydrate, ethyl acetoacetate, aldehyde, malononitrile	Fe_3_O_4_@THAM-piperazine	Water/EtOH	Heating (60 °C)	10–25 min	84–97%	Fatemeh Mir *et al.*^98^

### 4.2 Solvent-free synthesis

Solvent-free organic synthesis is an environmentally friendly approach that aims to reduce its ecological footprint by shunning conventional solvents and minimizing waste generation. This method boasts several notable characteristics, including shorter reaction times and simplified purification processes. These attributes confer several advantages, such as heightened safety, increased yield, and cost-effectiveness. Additionally, there is no need to evaporate solvents or heat them to overcome their boiling points.^99–101^

**Scheme 55:**
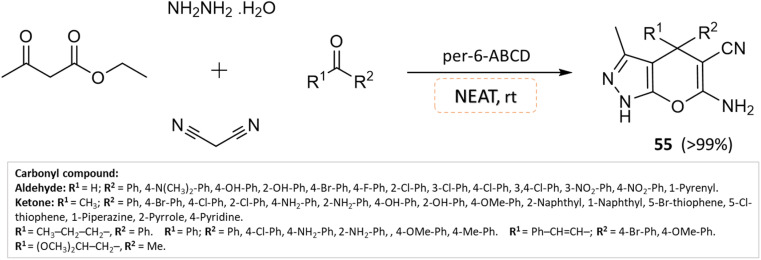
Synthesis of pyrano[2,3-*c*]pyrazole derivatives in solvent-free conditions using per-6-ABCD as the catalyst.

An effective solvent-free four-component synthesis of functionalized pyranopyrazoles was developed by Kanagaraj *et al.*^102^ using per-6-amino-β-cyclodextrin as the catalyst. A small amount of per-6-ABCD (0.008 mmol) combined with hydrazine hydrate, ethyl acetoacetate, aldehyde/ketone, and malononitrile yields quantitative yields of pyranopyrazoles in about a minute under solvent-free conditions (Scheme 55). This solid base catalyst can be reused six times without losing any of its catalytic activity.

**Scheme 56:**
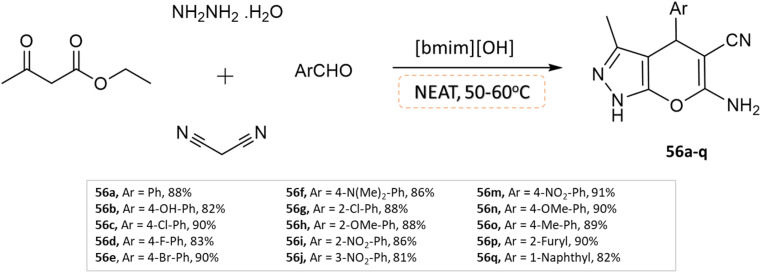
Synthesis of pyrano[2,3-*c*]pyrazole derivatives in solvent-free conditions using [bmim]OH.

Khurana and Chaudhary^103^ developed 4*H*-pyrano[2,3-*c*]pyrazoles using [bmim]OH as a task-specific ionic liquid *via* four-component condensation of aldehydes, malononitrile, ethyl acetoacetate, and hydrazine monohydrate without any use of a solvent (Scheme 56).

**Scheme 57:**
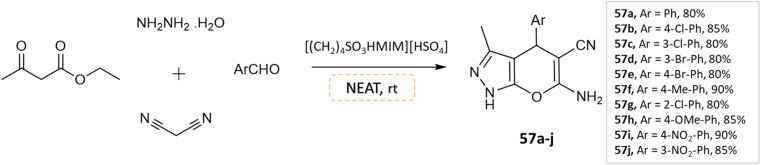
Synthesis of pyrano[2,3-*c*]pyrazole derivatives in solvent-free conditions using [(CH_2_)_4_SO_3_HMIM][HSO_4_].

J. Ebrahimi *et al.*^104^ reported the synthesis of biologically active pyranopyrazoles under a solvent-free environment *via* condensation of various aromatic aldehydes, ethyl acetoacetate, hydrazine hydrate, malononitrile in the presence of [(CH_2_)_4_SO_3_HMIM][HSO_4_], as an ionic liquid and catalyst (Scheme 57).

**Scheme 58:**
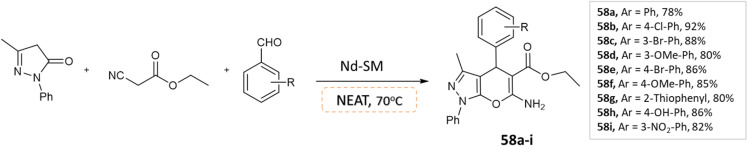
Synthesis of pyrano[2,3-*c*]pyrazole derivatives in solvent-free conditions using Nd-SM.

Abdullah Rather *et al.*^105^ reported a three-component one-pot synthesis of pyranopyrazole moieties *via* condensation of ethyl cyanoacetate, aldehyde, and pyrazolone under solvent-free conditions using a recyclable heterogeneous catalyst NdSM (Scheme 58). The study reports the application and synthesis of Nd-Salen immobilized mesoporous silica (NdSM) as a catalyst.

**Scheme 59:**
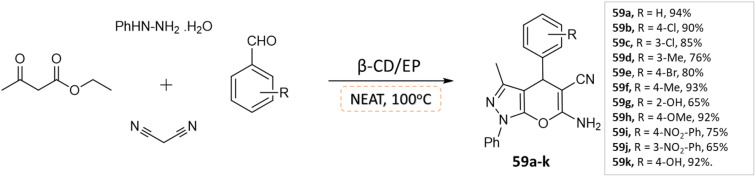
Synthesis of pyrano[2,3-*c*]pyrazole derivatives in solvent-free conditions using β-CD/EP.

**Scheme 60:**
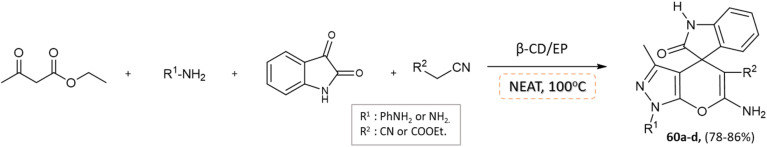
Synthesis of spiro-pyrano[2,3-*c*]pyrazole derivatives in solvent-free conditions.

J. Nasab and colleagues^106^ developed spiro[indoline-3,4′-pyrano(2,3-*c*)pyrazole] and pyranopyrazole derivatives using a water-insoluble nanosponge polymer called β-cyclodextrin/epichlorohydrin as a catalyst and a fixed micro-vessel under solvent-free thermal conditions. A mixture of aromatic aldehyde, phenylhydrazine, ethyl acetoacetate, malononitrile, and β-CD/EP was heated at 100 °C without any solvent for the pyranopyrazole synthesis, facilitated by the β-CD/EP catalyst (Scheme 59). For the synthesis of spiro[indoline-3,4′-pyrano(2,3-*c*)pyrazole] derivatives, a solvent-free reaction was conducted by stirring a mixture of phenylhydrazine, malononitrile, isatin, ethyl acetoacetate, and β-CD/EP at 100 °C. The β-CD/EP catalyst served as a stationary micro-vessel, enabling the reaction to proceed (Scheme 60).

**Scheme 61:**
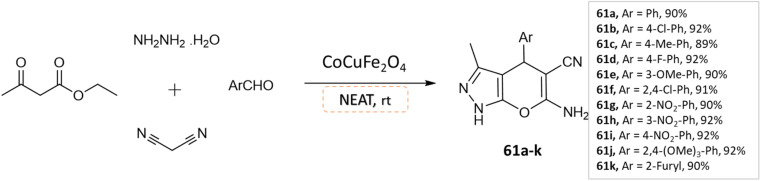
Synthesis of pyrano[2,3-*c*]pyrazole derivatives in solvent-free conditions using CoCuFe_2_O_4_ nanocrystals.

Dadaei and Naeimi^107^ reported the synthesis of pyrano[2,3-*c*]pyrazole derivatives at room temperature from ethyl acetoacetate, hydrazine hydrate, different aldehydes, and malononitrile, under a solvent-free environment in the presence of CoCuFe_2_O_4_ magnetic nanocrystals as a reusable catalyst (Scheme 61).

**Scheme 62:**
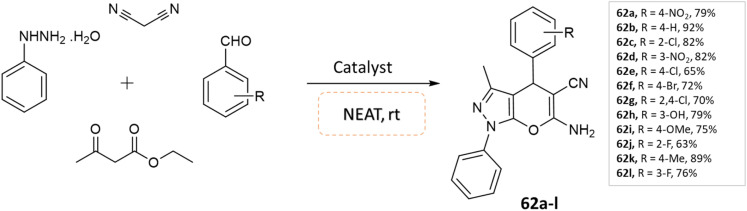
Synthesis of pyrano[2,3-*c*]pyrazole derivatives in solvent-free conditions using a nanomagnetic catalyst.

Soleimani and coworkers^108^ utilized Fe_3_O_4_@SiO_2_@Si(OEt)(CH_2_)_3_@melamine@TC@Cu(OAc)_2_ nanomagnetic catalyst under solvent-free conditions for the synthesis of pyrano[2,3-*c*]pyrazole derivatives. Reaction of phenylhydrazine, ethyl acetoacetate and benzaldehydes was carried out under the optimized reaction conditions in various solvents, namely ethyl acetate, acetonitrile, water, *n*-hexane, ethanol, and solvent-free condition. By examining the results of solvent-free conditions, it provides the shortest reaction time and the highest reaction yield. Additionally, the use of the magnetic catalyst significantly reduced the synthesis time of these compounds under solvent-free conditions (Scheme 62).

**Scheme 63:**
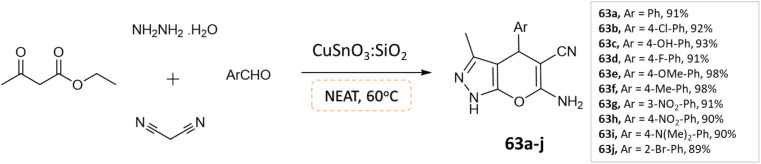
Synthesis of pyrano[2,3-*c*]pyrazole derivatives in solvent-free conditions using CuSnO_3_:SiO_2_ catalyst.

S. L. Sangle *et al.*^109^ utilized CuSnO_3_:SiO_2_ catalyst, synthesized using a hydrothermal method for the synthesis of pyranopyrazoles through a one-pot, four-component reaction involving aldehydes, malononitrile hydrazine hydrate, and ethylacetoacetate under solvent-free conditions (Scheme 63). This method demonstrated high yield and short reaction time, with an economically available catalyst and easy purification. The catalyst also showed potential as an alternative catalyst for various acidic-mediated reactions.

**Scheme 64:**
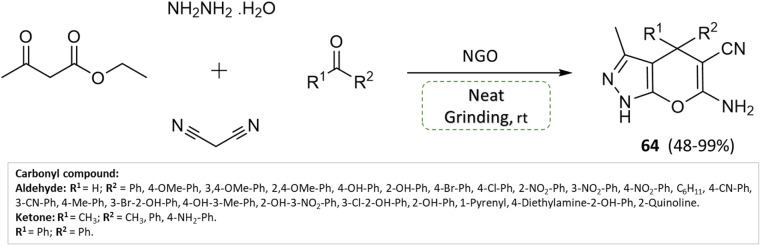
Synthesis of pyrano[2,3-*c*]pyrazole derivatives through neat grinding using nitrogen-doped graphene oxide.

S. Ganesan and P. Suresh^110^ hydrothermally synthesized nitrogen-doped graphene oxide (NGO) and investigated its application as a solid-base heterogeneous catalyst for pyranopyrazoles synthesis. Malononitrile, ethyl acetoacetate, hydrazine hydrate, and different functional groups of substituted aldehydes were combined in a condensation reaction under solvent-free conditions using a grinding technique, yielding high yields of pyranopyrazoles within 2 min (Scheme 64). Notably, the catalyst material exhibited stability and could be recycled and reused for up to eight consecutive cycles with only a trivial decrease in efficiency.

**Scheme 65:**
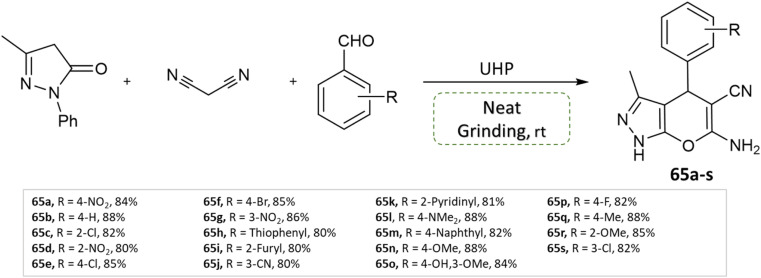
Synthesis of pyrano[2,3-*c*]pyrazole derivatives through neat grinding using urea hydrogen peroxide.

P. Verma *et al.*^111^ devised an eco-friendly approach devoid of metal catalysts, enabling the synthesis of a diverse array of dihydropyrano[2,3-*c*]pyrazole derivatives. This method involves 3-methyl pyrazolone, methyl arenes, malononitrile, and leverages urea hydrogen peroxide within a multicomponent reaction. Notably, this reaction is carried out under the solvent-free grinding method at ambient temperature (Scheme 65).

**Scheme 66:**
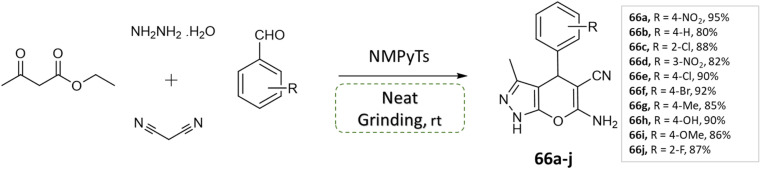
Synthesis of pyrano[2,3-*c*]pyrazole derivatives through neat grinding using ionic liquid (NMPyTs).

V. Sapkal *et al.*^112^ reported an efficient, green, and facile multi-component one-pot synthesis of pyrano[2,3-*c*] pyrazoles with various aryl aldehyde, malononitrile, ethyl acetoacetate, hydrazine hydrate under solvent-free grinding condition using ionic liquid (NMPyTs) as a catalyst (Scheme 66). A notable advantage of this protocol is its simplicity, solvent-free approach, easy workup, high yield, neat and clean synthesis (Table 7).

**Table 7:** A summary of solvent-free pyrano[2,3-*c*]pyrazoles and spiropyrano[2,3-*c*] pyrazoles synthesis

Product	Reactants	Catalyst	Solvent	Method employed	Reaction time	Yield	Ref.
Pyrano[2,3-*c*]pyrazoles	Four-component system: hydrazine hydrate, ethyl acetoacetate, aldehyde/ketone, and malononitrile	Per-6-amino-β-cyclodextrin	Solvent-free	Room temperature mixing	1–2 min	90–99%	Kanagaraj *et al.*^102^
4*H*-Pyrano[2,3-*c*]pyrazoles	Four-component system: aldehydes, malononitrile, ethyl acetoacetate, and hydrazine monohydrate	[bmim]OH ionic liquid	Solvent-free	Heating (50–60 °C)	5–10 min	81–91%	Khurana and Chaudhary^103^
Biologically active substituted pyranopyrazoles	Four-component system: aromatic aldehydes, ethyl acetoacetate, hydrazine hydrate, malononitrile	[(CH_2_)_4_SO_3_HMIM][HSO_4_]	Solvent-free	Room temperature stirring	30 min	80–90%	J. Ebrahimi *et al.*^104^
Pyrano[2,3-*c*]pyrazoles	Three-component system: aldehyde, ethyl cyanoacetate, and pyrazolone	Nd-Salen immobilized mesoporous silica (NdSM)	Solvent-free	Heating (70 °C)	10–20 min	78–92%	A. Rather *et al.*^105^
Pyranopyrazole derivatives	Four-component system: aromatic aldehyde, phenylhydrazine, ethyl acetoacetate, malononitrile	β-Cyclodextrin/epichlorohydrin (β-CD/EP)	Solvent-free	Heating (100 °C)	20–60 min	65–94%	J. Nasab *et al.*^106^
Pyrano[2,3-*c*]pyrazoles	Four-component system: ethyl acetoacetate, hydrazine hydrate, malononitrile, and aryl aldehydes	CoCuFe_2_O_4_ magnetic nanocrystals	Solvent-free	Room temperature stirring	40–45 min	89–92%	Dadaei and Naeimi^107^
Pyrano[2,3-*c*]pyrazoles	Four-component system: malononitrile, ethyl acetoacetate, phenyl hydrazine hydrate, and substituted aldehydes	Fe_3_O_4_@SiO_2_@Si(OEt)(CH_2_)_3_@melamine@TC@Cu(OAc)_2_	Solvent-free	Room temperature stirring	7–15 min	63–92%	Soleimani *et al.*^108^
Pyrano[2,3-*c*]pyrazoles	Four-component system: malononitrile, ethyl acetoacetate, hydrazine hydrate, and substituted aldehydes	CuSnO_3_:SiO_2_	Solvent-free	Heating (60 °C)	10 min	90–98%	S. L. Sangle *et al.*^109^
Pyrano[2,3-*c*]pyrazoles	Four-component system: malononitrile, ethyl acetoacetate, hydrazine hydrate, and substituted aldehydes	Nitrogen-doped graphene oxide (NGO)	Solvent-free (physical grinding)	Room temperature mixing	2 min	48–99%	S. Ganesan *et al.*^110^
Dihydropyrano[2,3-*c*]pyrazoles	Three-component system: methyl arenes, 3-methyl pyrazolone, and malononitrile	Urea hydrogen peroxide	Solvent-free (physical grinding)	Room temperature mixing	15–25 min	80–88%	P. Verma *et al.*^111^
Pyrano[2,3-*c*]pyrazoles	Four-component system: malononitrile, ethyl acetoacetate, hydrazine hydrate, and substituted aldehydes	NMPyTs (ionic liquid; boiled at 120 °C)	Solvent-free (physical grinding)	Room temperature mixing	10 min	80–95%	Amol V. Sapkal *et al.*^112^

The solvent-free approach offers numerous benefits, including cost-effectiveness, ease of purification, and excellent yields. It should be explored to optimize reaction time and other factors. Moreover, combining diverse green methodologies might enhance outcomes.

## 5 Conclusion

This review integrates several green methodologies that demonstrate their potential in the creation of structurally diverse and biologically relevant pyrano[2,3-*c*]pyrazole and spiro-pyrano[2,3-*c*]pyrazole derivatives. The article has analyzed both the notable benefits and constraints of these synthetic approaches to highlight forthcoming research directions, which prioritize safer reaction conditions, enhanced environmental factors, increased yields alongside improved selectivity, and the elimination of hazardous precursors, among other factors.

Techniques such as microwave heating, concentrated solar radiation, and ultrasound irradiation have emerged as rapid and energy-efficient alternatives to conventional heating. However, it is imperative that future investigations carefully consider scalability and reaction conditions to ensure practicality and effectiveness.

Catalytic strategies, including nano-catalysis, organo-catalysis, and bio-catalysis, have demonstrated unique advantages such as accelerated reactions and improved selectivity, all while maintaining an eco-friendly profile. Nevertheless, sustainable catalyst synthesis and a deeper understanding of their mechanisms are essential to maximize their efficiency in pyranopyrazole synthesis.

The pivotal role of water as a green solvent cannot be overstated, as it enables milder reaction conditions and reduces reliance on toxic organic solvents. Additionally, solvent-free synthesis aligns seamlessly with green principles, offering better results and cost-effective reactions. Exploring emerging green solvents with a keen focus on reaction parameters holds great potential for the synthesis of novel pyranopyrazole derivatives.

While significant strides have been made in the development of green multicomponent reactions for pyranopyrazole synthesis, there are still ample opportunities for further exploration and advancements. We hope that this comprehensive review article not only serves as a catalyst for further research but also inspires researchers to adopt these green approaches, leading to cleaner and more sustainable processes.

## Author contributions

Afrisham Ahmad: writing – original draft, conceptualization, Sithara Rao: formal analysis, validation, Nitinkumar S. Shetty: supervision, visualization.

## Conflicts of interest

There are no conflicts of interest to declare.

## Supplementary Material
